# Epigenetic variation between urban and rural populations of Darwin’s finches

**DOI:** 10.1186/s12862-017-1025-9

**Published:** 2017-08-24

**Authors:** Sabrina M. McNew, Daniel Beck, Ingrid Sadler-Riggleman, Sarah A. Knutie, Jennifer A. H. Koop, Dale H. Clayton, Michael K. Skinner

**Affiliations:** 1 Department of Biology, University of Utah, Salt Lake City, UT 84112-0840 USA; 2 Center for Reproductive Biology, School of Biological Sciences, Washington State University, Pullman, WA 99164-4236 USA

**Keywords:** Epigenetics, *Geospiza*, Copy number variation, Galápagos Islands, DNA methylation

## Abstract

**Background:**

The molecular basis of evolutionary change is assumed to be genetic variation. However, growing evidence suggests that epigenetic mechanisms, such as DNA methylation, may also be involved in rapid adaptation to new environments. An important first step in evaluating this hypothesis is to test for the presence of epigenetic variation between natural populations living under different environmental conditions.

**Results:**

In the current study we explored variation between populations of Darwin’s finches, which comprise one of the best-studied examples of adaptive radiation. We tested for morphological, genetic, and epigenetic differences between adjacent “urban” and “rural” populations of each of two species of ground finches, *Geospiza fortis* and *G. fuliginosa,* on Santa Cruz Island in the Galápagos. Using data collected from more than 1000 birds, we found significant morphological differences between populations of *G. fortis*, but not *G. fuliginosa*. We did not find large size copy number variation (CNV) genetic differences between populations of either species. However, other genetic variants were not investigated. In contrast, we did find dramatic epigenetic differences between the urban and rural populations of both species, based on DNA methylation analysis. We explored genomic features and gene associations of the differentially DNA methylated regions (DMR), as well as their possible functional significance.

**Conclusions:**

In summary, our study documents local population epigenetic variation within each of two species of Darwin’s finches.

**Electronic supplementary material:**

The online version of this article (doi:10.1186/s12862-017-1025-9) contains supplementary material, which is available to authorized users.

## Background

Studies of the molecular basis of evolutionary change have focused almost exclusively on genetic mechanisms. However, recent work suggests that heritable modifications to gene expression and function, independent of changes to DNA sequence, may also be involved in the evolution of phenotypes [1–3]. One of the most common of these epigenetic mechanisms is DNA methylation, i.e. the chemical attachment of methyl groups (CH_3_) to nucleotides (usually a cytosine followed by a guanine- “CpG”) [4]. Methylation can be induced by the environment and affect gene expression and phenotypic traits without changing the DNA sequence itself [5–8]. Importantly, some patterns of methylation are heritable, meaning they have the potential to evolve [9–14]. Indeed, because DNA methylation modifications (epimutations) are more common than genetic mutations [15], they may play a role in the rapid adaptation of individuals to new or variable environments [16].

Environmentally-induced epimutations may be a component of the adaptive radiation of closely related species to new environments [17]. For example, Skinner et al. [18] showed that epigenetic variation is significantly correlated with phylogenetic distance among five closely related species of Darwin’s finches in the Galápagos Islands. Although the adaptive significance of this epigenetic variation is unknown, some of the variants are associated with genes related to beak morphology, cell signaling, and melanogensis. The results of this study suggest that epigenetic changes accumulate over macroevolutionary time and further suggest that epigenetic changes may contribute to the evolution of adaptive phenotypes.

Epigenetic variation also occurs among populations within single species [15, 19–24]. Some population epigenetic studies report correlations between methylation patterns and environmental factors, suggesting that differences in methylation are involved in local adaptation to different environments [21, 22, 24]. For example, in a study of populations of two salt marsh specialist plants living along a salinity gradient, Foust et al. [24] found that ground salinity is more closely correlated with epigenetic variation than genetic variation.

**Fig. 1:**
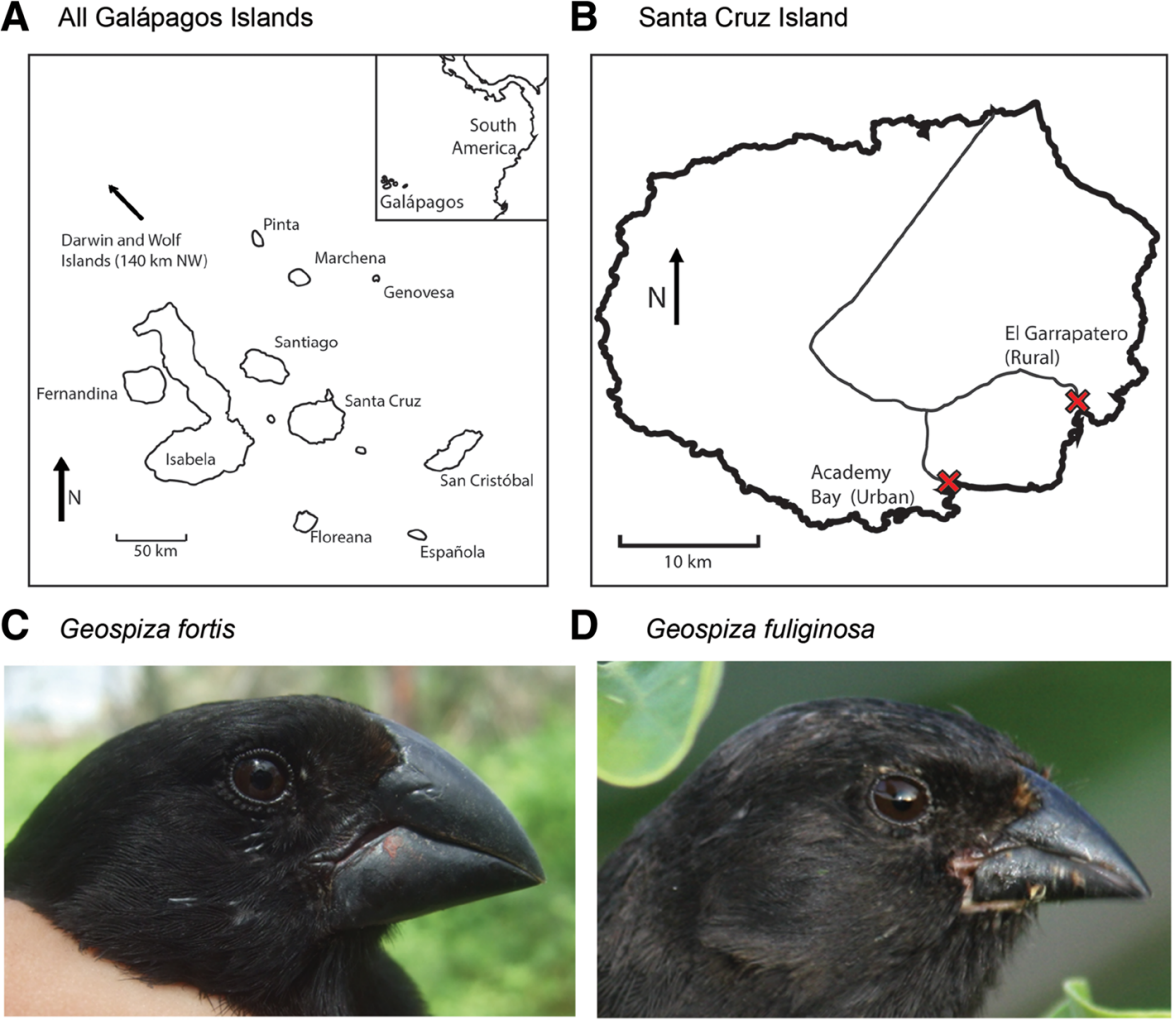
Study sites and species. **a** The Galápagos Archipelago. **b** Santa Cruz Island; Roads are indicated by *narrow grey lines* and study sites by *red* Xs. **c** *Geospiza fortis*; photo by J.A.H.K. **d** *Geospiza fuliginosa*; photo by S.A.K. Maps in (**a**) and (**b**) are modified from © 2016 Google

The purpose of our study was to investigate epigenetic variation between populations of each of two species of Darwin’s finches: the medium ground finch *(Geospiza fortis)* and the small ground finch *(G. fuliginosa)* (Fig. 1). Darwin’s finches are a closely related group of about 16 species endemic to the Galápagos Islands [25–28]. Long-term studies show rapid phenotypic changes in populations of finches in response to environmental pressures, including competition [26]. The molecular basis of these phenotypic changes is poorly known. Although recent genomic studies have identified alleles in several putative genes associated with beak size and shape [28–30], most genetic markers show little differentiation among populations or species [28, 30–34].

Epigenetic variation may contribute to the phenotypic diversity of Darwin’s finch populations that cannot be detected through genomic studies. As an initial test of this hypothesis, we compared components of the morphology, genetics, and epigentics in populations of finches living at El Garrapatero, a relatively undisturbed locality, to populations living near Puerto Ayora (Academy Bay), the largest town in the Galápagos Islands (Fig. 1). Hereafter, we refer to these as the “rural” and “urban” sites, respectively. The two sites, which are only 10 km apart, are both arid, lowland scrub habitat along the south and south-eastern coast of the island. Vegetative cover, based on remote sensing spectroradiometric indicies, is slightly higher at the urban site; however, cover is highly correlated between the two sites year-round (Additional file 1: Figure S1). Despite the overall ecological similarity of the sites, anthropogenic disturbance at the urban site has increased dramatically over the past fifty years [35]. Observational studies suggest urbanization has effects on finch behavior and diet: birds in the urban population incorporate novel, human foods into their diets, whereas finches in the rural popuation do not [36]. To further explore potential impacts of urbanization of Puerto Ayora on ground finches, we tested for morphological, genetic, and epigenetic differences between urban and rural populations in each of two species of finches.

## Methods

### Study sites and species

We studied each of two populations of *G. fortis* and *G. fuliginosa* living in urban and rural environments (urban: Academy Bay; 0° 44′ 21.3″ S, 90° 18 ‘06.3″ W; rural: El Garrapatero; 0° 41′ 15.7″ S, 90° 13′ 18.3″ W). The two localities, which are separated by about 10 km, are both in the arid coastal zone of Santa Cruz Island (Fig. 1). *Geospiza fortis* and *G. fuliginosa* are among the most abundant species of finches at these study sites. There appears to be little movement of finches between populations. Over the course of a decade-long banding study (2002–2012), during which more than 3700 finches were captured- and more than 300 individuals recaptured- only one bird (a female *G. fortis*) was shown to have moved between the two sites (J. Raeymaekers pers. comm.).

### Field work and sample collection

Finches were captured at the two study sites January–April 2008–2016. The birds were mist-netted and banded with individually numbered Monel bands in order to track individuals. They were aged and sexed using size and plumage characteristics [37]. Morphological measurements were taken from each individual including beak depth, beak width, beak length, tarsus length, wing chord, and body mass, following Grant and Grant (2014) [26], with the exception that wing chord was measured unflattened. Principle components were calculated from untransformed data for the three body measurements (mass, wing chord, and tarsus) and for the three beak measurements (length, width, and depth) to provide aggregate measures of body size and beak size and shape [38]. We evaluated morphological differences between urban and rural sites using linear mixed effects models (LMM), with site as a fixed effect, and year as a random effect to control for variation among years and investigators. Separate models were run for each morphological measurement, as well as body size (PC1 body) and beak size and shape (PC1 beak and PC2 beak). *P-*values were adjusted with a Bonferroni correction for multiple tests. Morphological analyses were run in the program RStudio using R version 3.2.1 with the packages pwr, plotrix, lme4, and lmerTest [39–42].

Blood and sperm samples for epigenetic and genetic analyses were collected from a subset of birds captured January–April 2009–2013 at the two study sites. Blood samples (<90 μl) were taken from finches via brachial venipuncture. The samples were stored on wet ice in the field and, within six hours of collection, erythrocytes were purified by centrifugation. Sperm samples (~5 μl) were taken from a subset of males. The sperm samples were obtained by gentle squeezing of the cloacal protuberance of reproductively active males. Blood erythrocytes and sperm samples were stored in a − 20 °C freezer in the Galápagos. Following each field season, they were transferred to a − 80 °C freezer in the USA for long-term storage. All field procedures were approved by the University of Utah Institutional Animal Care and Use Committee (protocols #07–08004, #10–07003 and #13–06010) and by the Galápagos National Park.

### Genomic DNA preparation

Genomic DNA from finch red blood cells (erythrocytes) was prepared using the Qiagen DNAeasy Blood and Tissue Kit (Qiagen, Valencia CA). The manufacturer’s instructions for nucleated blood samples were followed, but in the final DNA elution step H_2_O was used instead of the buffer provided in the kit. Genomic DNA from finch sperm was prepared as follows: collected sperm suspension was adjusted to 100 μl with 1 x Phosphate Buffered Saline (PBS) then 820 μl DNA extraction buffer (50 mM Tris pH 8, 10 mM EDTA pH 8, 0.5% SDS) and 80 μl 0.1 M dithiothreitol (DTT) were added and the sample was incubated at 65 °C for 15 min. Next, 80 μl Proteinase K (20 mg/ml) were added and the sample was incubated on a rotator at 55 °C for 2 h. After incubation, 300 μl of protein precipitation solution (Promega, A795A) were added, then the sample was mixed and incubated on ice for 15 min, then spun at 4 °C at 13,000 rpm for 20 min. The supernatant was transferred to a fresh tube, then precipitated over night with the same volume of 100% isopropanol and 2 μl glycoblue at −20 °C. The sample was then centrifuged and the pellet washed with 75% ethanol, then air-dried and re-suspended in 100 μl H_2_O. DNA concentration was measured using a Nanodrop Spectrophotometer (Thermo Fisher).

### CNV-Seq protocol

To test for genetic differences between the urban and rural populations we sequenced DNA extracted from red blood cells (erythrocytes) and compared genetic copy number variation (CNV) [18]. CNV, defined as the changes in the number of repeat element copies of more than >1 kb of DNA, is increasingly recognized as one of the most common and functionally important markers of genetic variation [43]. The basic copy number variation (CNV) was determined through genomic sequencing of the same samples used for epigenetic analysis. Read numbers at specific loci were compared genome wide to identify CNV [18]. Erythrocyte DNA pools were generated by combining equal amounts of extracted DNA from five individuals. Each pool contained a total of 2 μg of genomic DNA. Three pools of five individuals each were created per species, per site. Pooling samples for genomic analysis provides an accurate and cost-effective way of comparing populations [44]. Pooling decreases power, compared to sequencing individual samples. Although minor differences in copy number between populations may be missed [45], large differences between groups should be detected.

The pools were diluted to 130 μl with 1 x TE buffer and sonicated in a Covaris M220 with the manufacturer’s preset program to create fragments with a peak at 300 bp. Aliquots of the pools were run on a 1.5% agarose gel to confirm fragmentation. The NEBNext DNA Library Kit for Illumina was used to create libraries for each pool, with each pool receiving a separate index primer. The libraries were sent to the University of Nevada, Reno Genomics Core for NGS on the Illumina HiSeq 2500 using a paired end PE50 application. All 6 pooled sequencing libraries for each species were run in one sequencing lane to generate approximately 30 million reads per pool. The read depth across the genome was then assessed to identify CNV and statistically assessed with a Bayesian analysis. The genome-wide paired end read depth was approximately 2× with the CNV read depth being a total of 300 to 6000 reads per CNV detected.

### Methylated DNA Immunoprecipitation (MeDIP)

Following Skinner et al. [18], we used erythrocytes as a purified somatic cell type to compare differentially methylated regions (DMRs) between populations of each of the two species. For a subset of birds, we also compared DMR of germ line cells (sperm). DMRs between urban and rural populations were identified by the methylated DNA immunoprecipitation (MeDIP) of genomic DNA. MeDIP is an enrichment-based technique that uses an antibody to preferentially precipitate methylated regions of the genome that are then sequenced [46]. DMRs are identified by comparing coverage between groups of interest. MeDIP is a cost-effective way to evaluate genomic CpG methylation, and provides highly concordant results to other sequencing-based DNA methylation methods, such as bisulfite sequencing [47]. Because MeDIP surveys methylation genome-wide, it can be used to identify genomic characteristics associated with methylation. For instance, studies have found relationships between CpG density, methylation, and effects on gene transcription [6].

For analysis of erythrocytes, genomic DNA was extracted from the same individuals as used in the CNV pools. Each erythrocyte pool included five individuals and contained a total of 6 μg of genomic DNA. Sperm pools included two individuals and contained a total of 1.8 μg of genomic DNA. Three pools were generated per species, per site (for a total of *n* = 6 individuals per species, per site for sperm and *n* = 15 individuals per species per site for erythrocytes to consider biological variation of the pools and analysis). All pools were diluted to 150 μl with 1× Tris-EDTA (TE, 10 mM Tris, 1 mM EDTA) and sonicated with a probe sonicator using 5 × 20 pulses at 20% amplitude. Fragment size (200–800 bp) was verified on 1.5% agarose gel. Sonicated DNA was diluted to 400 μl with 1xTE and heated to 95 °C for 10 min, then shocked in ice water for 10 min. Next, 100 μl of 5 x immunoprecipitation (IP) buffer (50 mM Sodium Phosphate pH 7, 700 mM NaCl, 0.25% Triton X-100) and 5 μg of 5-mC monoclonal antibody (Diagenode, C15200006–500) were added and the sample was incubated on a rotator at 4 °C over night. The next day Protein A/G Agarose Beads from Santa Cruz Biotechnology, Santa Cruz CA, were prewashed with 1xPBS/0.1% BSA and re-suspended in 1 x IP buffer. Eighty μl of the bead slurry were added to each sample and incubated at 4 °C for 2 h on a rotator. The bead-DNA-antibody complex was washed 3 times with 1 x IP buffer by centrifuging at 6000 rpm for 2 min and re-suspending in 1 x IP buffer. After the last wash the bead-complex was re-suspended in 250 μl of digestion buffer (50 mM Tris pH 8, 10 mM EDTA pH 8, 0.5% SDS) with 3.5 μl Proteinase K (20 mg/ml) per sample and incubated on a rotator at 55 °C for 2 h. After incubation, DNA was extracted with the same volume of Phenol-Chloroform-Isoamyalcohol, then with the same volume of chloroform. To the supernatant from chloroform extraction, 2 μl glycoblue, 20 μl 5 M sodium chloride and 500 μl 100% cold ethanol were added. DNA was precipitated at −20 °C over night, then spun for 20 min at 13,000 rpm at 4 °C, washed with 75% ethanol, and air-dried. The dry pellet was re-suspended in 20 μl H_2_O and concentration measured in Qubit using a Qubit ssDNA Assay Kit (Life Technologies, Carlsbad, CA).

### MeDIP-Seq protocol

The next step for DMR identification involved sequencing the MeDIP DNA to identify differential methylation at specific genomic loci by assessing read numbers for the different samples. The MeDIP pools were used to create sequencing libraries for next generation sequencing (NGS) at the University of Nevada, Reno Genomics Core Laboratory using the NEBNext® Ultra™ RNA Library Prep Kit for Illumina®, starting at step 1.4 of the manufacturer’s protocol to generate double stranded DNA. After this step the manufacturer’s protocol was followed. Each pool received a separate index primer. NGS was performed at the same laboratory using the Illumina HiSeq 2500 with a paired end PE50 application, with a read size of approximately 50 bp and approximately 100 million reads per pool. Two separate sequencing libraries, one rural and one urban, were run in each lane. The read depth for identified differential DNA methylated regions (DMRs) ranged from approximately 100 to >1000 total reads per DMR.

### Bioinformatics

Basic read quality was verified using summaries produced by the FastQC program [48]. The reads for each sample for both CNV and DMR analyses were mapped to the zebra finch (*Taenopygia guttata*) genome using Bowtie2 [49] with default parameter options. The mapped read files were converted to sorted BAM files using SAMtools [50]. The cn.MOPS R package [51] was used to identify potential CNV. The cn.mops default information gain thresholds were used for this analysis. The cn.MOPS analysis detects CNVs by modeling read depth across all samples. The model predicts copy number for a given window based on observed read counts. The model uses a Bayesian framework to determine whether copy number for a give window differs significantly from 2. The length of the CNV is determined by comparing copy number of adjacent windows on the genome and joining those with the same copy number into one segment. A CNV call occurs when copy number for a given genomic segment varies from that of other samples. CNV detection with cn.MOPS is robust to low-coverage sequencing data (0.18–0.46 for 75 bp reads) and performs well when comparing 6 or more samples [51]. The window size used by the cn.MOPS analysis was chosen dynamically for each chromosome based on the read coverage. The chromosomes’ window size ranged from approximately 25 kb to 60 kb. Only CNV that occurred in either all urban or all rural pools were compared. Although some individual pools had higher numbers of CNV, only CNV that occur red among all the pools were included in the analysis. The CNV are identified using the difference between the posterior and prior distributions from the Bayesian analysis to estimate information gain.

To identify DMR, the reference genome was broken into 100 bp windows. The MEDIPS R package [52] was used to calculate differential coverage between the urban and rural localities. The edgeR *p*-value [53] was used to determine the relative difference between the two localities for each genomic window. Windows with an edgeR *p*-value less than 10^−3^ were considered DMR. The DMR edges were extended until no genomic window with an edgeR *p*-value less than 0.1 remained within 1000 bp of the DMR. The DMR that included at least two windows with an edgeR *p*-value <10^−3^ (“multiple-window DMR”) were then selected for further analysis. Because no fully assembled or annotated genome exists for any Darwin’s finch species, we aligned DMR with the zebra finch genome. CpG density and gene associations were then calculated for the DMR, based on alignment with the reference genome. Though we previously found high (>98%) homology between Darwin’s finch and zebra finch genomes using tiling arrays [18], some differences were expected. Thus, associations of DMR with genes are likely to be under-estimates. To validate the epigenetics and gene associations, a similar analysis was also done with the draft *G. fortis* genome [54]. All the DMR sequence and genomic data obtained in the current study have been deposited in the NCBI public GEO database (GEO # GSE87825).

DMR clusters were identified with an in-house R script (www.skinner.wsu.edu under genomic data) using a 2 Mb sliding window with 50 kb intervals. DMR were annotated using the biomaRt R package [55] to access the Ensembl database [56]. The genes that overlapped with DMR were then input into the KEGG pathway search [57, 58] to identify associated pathways. A 10 kb flanking sequence was added to each DMR to consider potential localization in promoter regions of the gene as previously described [18, 59]. The DMR associated genes were manually sorted into gene classification groups by consulting information provided by the DAVID, Panther, and Uniprot databases incorporated into an internal curated database (www.skinner.wsu.edu under genomic data). To assess that the DMR were not false positives due to random biological variation within populations, a pairwise comparison analysis (individual pool comparison) on the genomic sequence data within the individual urban or rural sites and cell populations was performed [60].

## Results

### Morphology

We used 1097 birds captured between 2008 and 2016 for morphological analyses. We controlled for slight variation among years in traits by including year as a random effect in all analyses. At both sites, *G. fortis* was significantly larger than *G. fuliginosa* in all morphological traits (linear mixed-effects models *P* < 0.0001). Within species, urban *G. fortis* was significantly larger than rural *G. fortis* for all direct morphological measurements, except tarsus length (Table 1). Composite measures of *G. fortis* body and beak size (PC1 body and PC1 beak) also differed between the two sites; however, there was no difference in beak shape (PC2 beak). In contrast to *G. fortis*, *G. fuliginosa* did not differ significantly between the urban and rural populations in any of the morphological measurements or composite measures (Table 1). Because we captured more *G. fortis* than *G. fuliginosa* we did a power analysis for *G. fuliginosa*, using the effect size of morphological differences found in the *G. fortis* populations (smallest effect size = 0.256 (wing chord); largest effect size = 0.358 (beak depth)). Power for comparisons of *G. fuliginosa* appeared adequate for detecting similar effect sizes (0.69–0.91).

**Table 1:** Mean (± 1SE) values for morphological characteristics of *G. fortis* and *G. fuliginosa* at rural vs. urban sites.

	*G. fortis*		*G. fuliginosa*	
Morphological	Rural	Urban	Rural	Urban
Character	*N* = 560	*N* = 245	*N* = 171	*N* = 121
Beak depth	11.48 ± 0.06	11.98 ± 0.09**	7.40 ± 0.04	7.42 ± 0.06
Beak width	9.89 ± 0.04	10.24 ± 0.07**	6.8 ± 0.03	6.82 ± 0.04
Beak length	11.71 ± 0.04	12.02 ± 0.07***	8.56 ± 0.04	8.46 ± 0.09
Tarsus length	21.00 ± 0.06	21.15 ± 0.09	18.83 ± 0.11	18.67 ± 0.09
Wing chord	69.3 ± 0.19	70.4 ± 0.29**	61.26 ± 0.31	61.1 ± 0.30
Body mass	21.23 ± 0.13	22.2 ± 0.23*	13.87 ± 0.15	13.76 ± 0.14
PC1 Body	−0.13 ± 0.06	0.29 ± 0.09***	0.07 ± 0.09	−0.10 ± 0.10
PC1 Beak	−0.17 ± 0.07	0.40 ± 0.11***	0.01 ± 0.09	−0.01 ± 0.15
PC2 Beak	−0.01 ± 0.02	0.02 ± 0.03	0.07 ± 0.04	−0.09 ± 0.10

Statistically significant differences between populations at *P* < 0.01, 0.001, and <0.0001 are indicated by *, ** and ***, respectively

### Copy number variation (CNV)

Mean read depth genome-wide for pools used in CNV analysis varied between 1.08× and 1.30× (overall mean = 1.22×). The total read depth of individual variants ranged from 300 to 6000. We identified unique CNV in three of six *G. fortis* pools and five of six *G. fuliginosa* pools. The total number of variants per pool ranged from 1 to 20. However, no variants were exclusive to all urban or all rural pools for either *G. fortis* or *G. fuliginosa* (Additional file 2: Fig. S2). Therefore, while there was variation within populations in copy number at various loci in both *G. fortis* and *G. fuliginosa* (e.g., FB2 & 12), there were no fixed differences between the urban and rural populations for either species. It is unclear why certain pools had more variants than others; however variation was consistent among chromosomes.

To control for underestimation of CNV differences due to reads that did not align to the zebra finch genome, we performed a similar analysis aligning reads to the un-assembled *Geospiza fortis* genome [54]. The average proportion of reads aligned to the *G. fortis* genome was higher (two-fold). However, we still did not find any differences in CNV between the urban and rural populations for either species of Darwin’s finch. A limitation of this CNV analysis is that only large variants (>24 Kbp) can be detected reliably; smaller variants (<10 Kbp or less) may have escaped detection.

### Differential DNA methylation regions (DMRs)

**Fig. 2:**
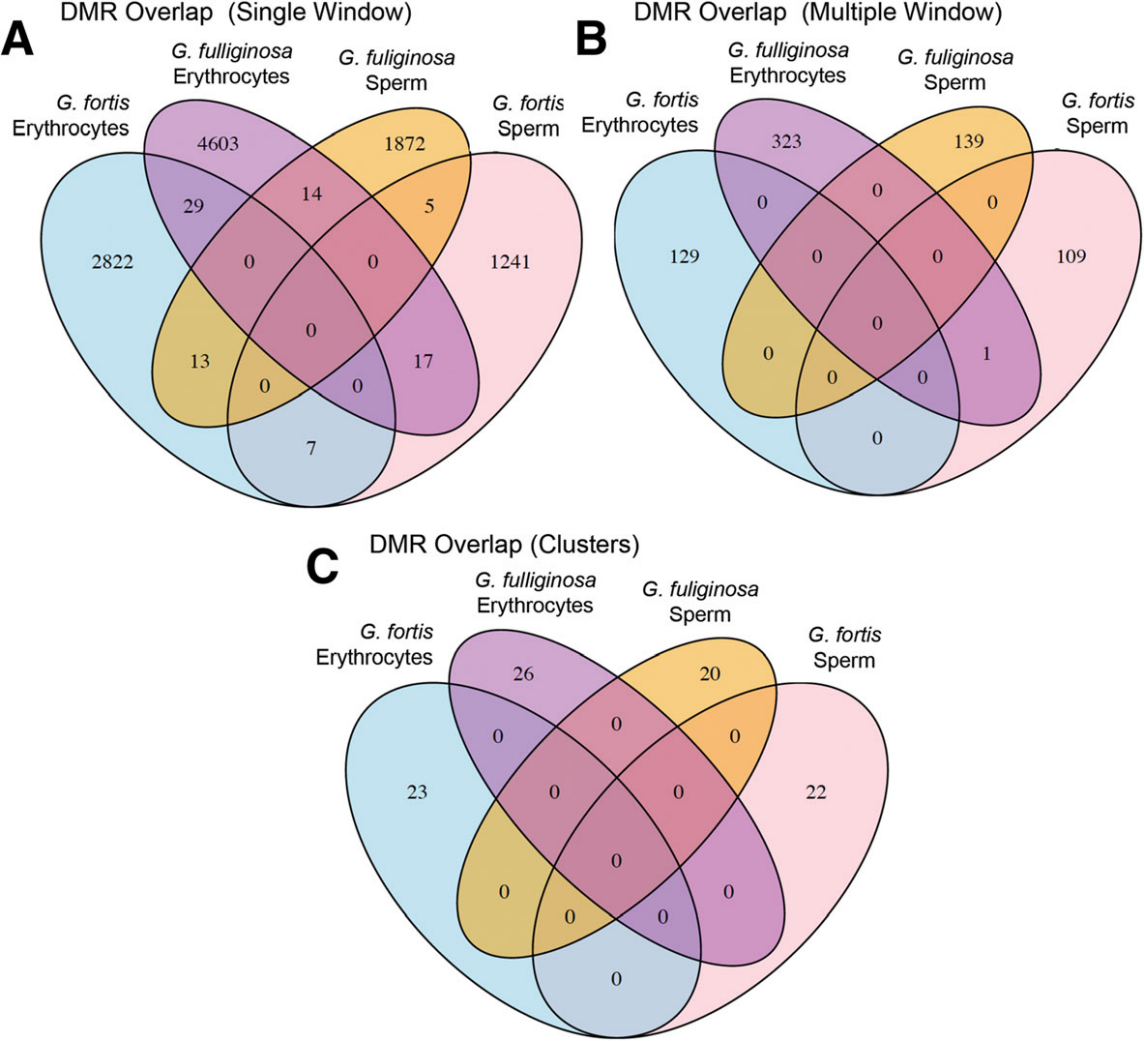
DMR overlap between species and cell types. Each value is the number of differentially methylated regions between the urban and rural populations. Overlapping colors in the figure show the number of DMR that are shared between the two species or the two cell types. DMRs detected within **a** single 100 bp windows, **b** 2–5 adjacent 100 bp windows, **c** 2–7 Mb regions

DMRs were found between populations for both cell types and both species (Table 2). We report the number of DMRs at *p*-value cut-offs ranging from <0.01 to <1e-05 in Additional file 3: Table S1; Additional file 4: Table S2 and Additional file 5: Table S3. The analyses reported below are restricted to DMRs significant at a level of *P* < 0.001. We evaluated differences on three “regional” scales (Fig. 2): single 100 bp window DMRs, multiple window DMRs, and “DMR clusters”, i.e. statistically over-represented DMR clusters of 3–10 DMRs spanning 2–7 Mb [18] (Additional file 6: Table S4A-D). We focus on multiple-window DMRs (Additional file 4: Table S2 and Additional file 5: Table S3), i.e. DMRs detected independently in adjacent windows, because they further reduce the likelihood of false positives and provide a set of highly reproducible DMRs [18]. Multiple-window DMRs were used in the analysis of the genomic features of DMRs reported below.

**Table 2:** Differentially methylated regions (DMR) between urban and rural populations based on different cell types

Species/Cell Type	Number of Windows*					Sum of Multiple (≥2) Window DMR**
	1	2	3	4	5	
*G. fortis* Erythrocytes	2742	125	4	0	0	129
*G. fortis* Sperm	1160	97	9	3	1	110
*G. fuliginosa* Erythrocytes	4339	314	9	1	0	324
*G. fuliginosa* Sperm	1765	133	6	0	0	139

Only DMR that were significant at *P* < 0.001 are included

*DMR detected in one window alone were considered “single-window” variants (Fig. 2)

**Fig. 3:**
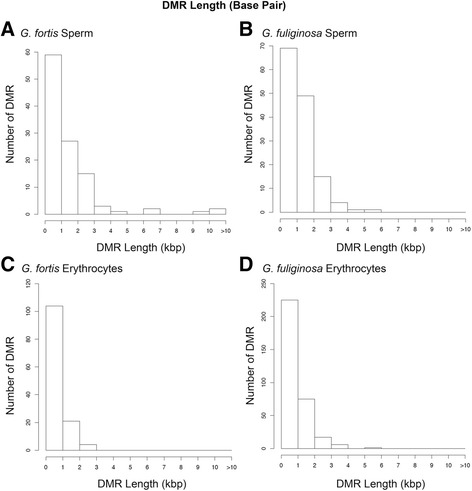
DMR length (kb) in **a** *G. fortis* sperm. **b** *G. fuliginosa* sperm. **c** *G. fortis* erythrocytes. **d** *G. fuliginosa* erythrocytes. Only multiple-window DMR significant at a *p*-value threshold of <10^−3^ are included

**Fig. 4:**
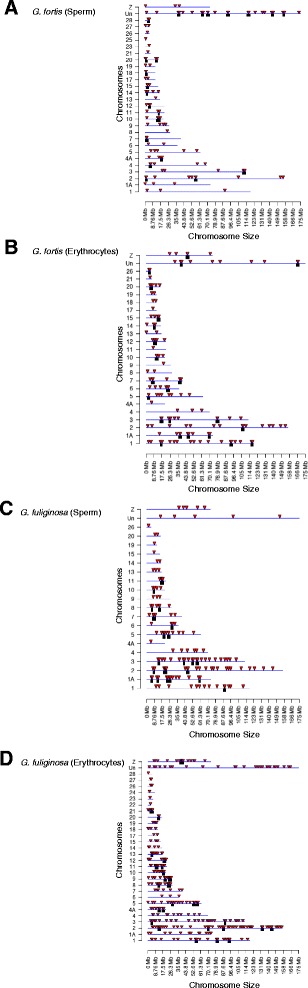
Chromosomal locations of DMR identified in *Geospiza fortis* sperm **a** and erythrocytes (**b**) and *G. fuliginosa* sperm (**c**) and erythrocytes (**d**)**.** Locations are based on alignment to the zebra finch (*Taeniopygia guttata*) genome. *Red arrowheads* indicate DMR and *black boxes* indicate DMR clusters. Only multiple-window DMR significant at a *p*-value threshold of <10^−3^ are shown

**Fig. 5:**
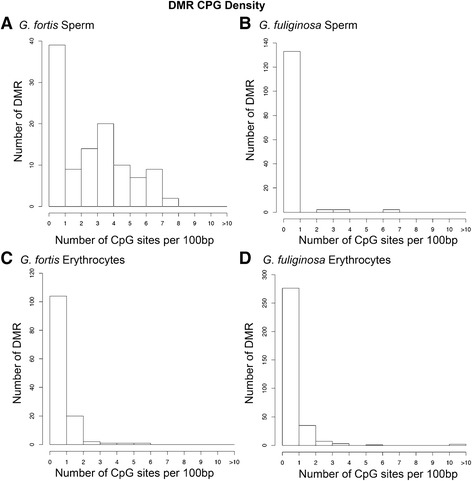
The CpG density of DMR in *Geospiza fortis* sperm (**a**), *G. fuliginosa* sperm (**b**), *G. fortis* erythrocytes (**c**) and *G. fuliginosa* erythrocytes (**d**). Only multiple-window DMR significant at a *p*-value threshold of <10^−3^ are included

**Fig. 6:**
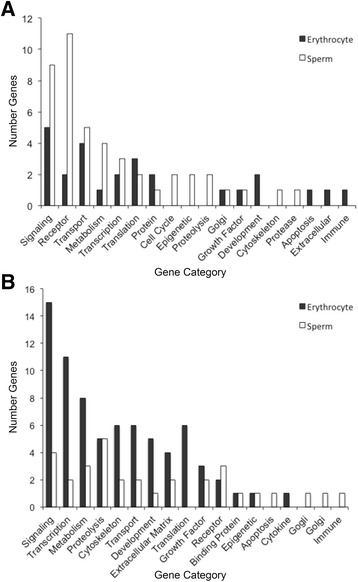
Gene categories associated with DMR detected in (**a**) *G. fortis* and (**b**) *G. fuliginosa*. Only multiple-window DMR significant at a *p*-value threshold of <10^−3^ are included

**DMR detected in two or more adjacent windows were considered “multiple-window” variants and used in subsequent analyses (Figs. 2, 3, 4, 5 and 6)

There was little overlap between species or cell types in the regions that were differentially methylated between urban and rural populations (Fig. 2). A small proportion of single window DMRs (Fig. 2A) was shared between species and/or cell types. However, there were virtually no shared multiple-window DMRs (Fig. 2B) or clusters of DMRs (Fig. 2C) between species and/or cell types*.*

For both species and cell types, multiple-window DMRs usually were detected in only two multiple 100 bp windows; however, a limited number (<10% of total DMRs) were found in 3–5 multiple windows (Table 2). Based on extension of edges of multiple-window DMRs (extension of adjacent 100 bp windows with *p* < 0.1; see Methods) we estimated that most DMRs were 500–1000 bp in length (Fig. 3). Many of the DMRs in this study were clustered together, consistent with previous studies showing that DMRs are not evenly distributed across the genome [59]. Based on alignment to the zebra finch genome, we plotted the chromosomal locations of multiple-window DMRs and DMR clusters (Fig. 4). DMRs were present on all chromosomes in both sperm and erythrocytes of both species; however, the chromosomal locations of DMRs differed between the cell types and species.

We evaluated the location of DMRs with respect to nucleotide composition. CpG density was highest in DMRs of *G. fortis* sperm cells (Fig. 5A). DMRs in *G. fortis* erythrocytes and both cell types of *G. fuliginosa* were most often found in lower density CpG regions of the genome (<1 CpG site/100 bp; Fig. 5B-D). We estimated that the DMRs typically had approximately 10 CpG sites clustered within 1 kb regions.

We identified potential genes associated with DMRs through alignment with the zebra finch reference genome. DMRs within 10 kb of a gene (such that the promoter is included) have the potential to influence the gene’s expression and/or pathways associated with that gene [59]. Different categories of genes were methylated in the two cell types and species (Fig. 6, specific genes listed in Additional file 7: Table S5). The most common gene categories associated with DMRs were metabolism, cell signaling and transcription (Fig. 6). Gene categories associated with DMRs differed significantly between the two species (Chi-square test, *p* = 0.039) and marginally between the two cell types (Chi-square test; *p* = 0.078). Pathway analysis (KEGG) showed DMRs associated with several genes (GALNT14, SGMS1, ENO2, PLCH2) in metabolic pathways of *G. fortis* sperm. DMRs were associated with different genes (GCLC, PRIM2, ALD1A3, AK4, ACACA) in metabolic pathways of *G. fuliginosa* sperm. *Geospiza fortis* erythrocyte DMRs were associated with genes (CACNA1H, FGF8, MRAS, RAP1A) in the MAPK signaling pathway. *Geospiza fuliginosa* erythrocyte DMRs were not associated with any particular pathway.

**Fig. 7:**
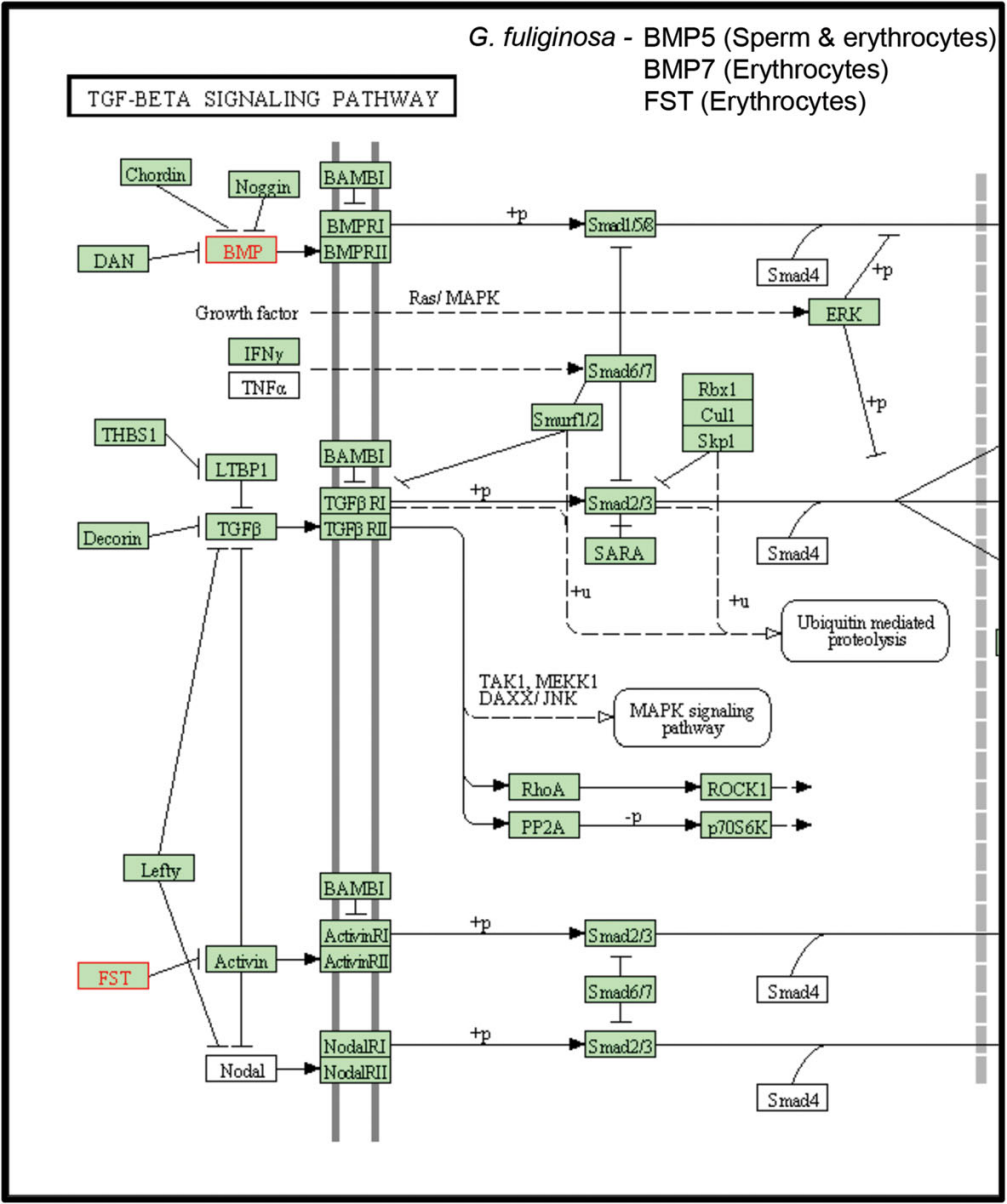
TGFB/BMP pathway. Genes associated with DMR are listed and outlined in *red* in the pathway

When the DMR data sets for both species and cell types were compared, KEGG pathways with the most DMR-associated genes were metabolic pathways, and MAPK and TGFß/BMP signaling pathways. Metabolic pathways included glycolysis, in which genes involved with pyruvate and acetate production were associated with DMRs in both finch species (Additional file 8: Figure S3 and Additional file 9: Figure S4). Other metabolic pathways associated with DMRs included genes involved in purine metabolism and glycosylation (Additional file 7: Tables S5). Signaling pathways were also associated with DMRs in both species and cell types. Three genes in the TGFß/BMP pathway were associated with DMRs between *G. fuliginosa* populations (erythrocytes and sperm combined): BMP5, BMP7 and FST (Fig. 7). MAPK, a common pathway for many regulatory processes, such as cell growth, contained a high number of DMR-associated genes in both finch species (Additional file 8: Figure S3 and Additional file 9: Figure S4).

Genomic correlates of our DMR and CNV data were analyzed using the well-annotated zebra finch genome. In addition, our sequencing data were also compared to the *G. fortis* shotgun sequence database [54]. In contrast to the zebra finch genome, the *G. fortis* genome is neither assembled, nor annotated, meaning that limited data analysis is possible. The pooled individual sample read number was approximately 100 million reads for both genome analyses. The overall read alignment rate was 47–48% for the zebra finch analysis and 70–75% for the *G. fortis* genome analysis. Although previous analysis using tiling arrays suggested a 98% similarity in tiling array hybridization of the genome [18], the next generation sequencing analysis shows that more differences exist, likely in non-coding regions. The zebra finch genome analysis revealed twice the number of DMRs compared to the *G. fortis* genome analysis. This was largely due to the incomplete nature of the *G. fortis* genome. Nevertheless, analysis with both the zebra finch and *G. fortis* genomes identified epigenetic alterations between the rural and urban sites. To test whether methylation variation between sites was greater than within sites we conducted a pairwise comparison analysis (comparison of individual pools) within each species and rural or urban populations for specific cell types. We identified a number of DMRs between individual pools, which suggests that there is epigenetic variation within the study populations. However, few DMRs were found in multiple pools from the same population. Moreover, almost none of these DMRs were also found between urban and rural populations (Additional file 10: Figure S5). Thus, the DMRs identified between urban and rural populations are not an artifact of sampling within-population variation.

## Discussion

Darwin’s finches are well known for their phenotypic variability and evolution in response to changing environmental conditions [26]. In addition to genetic variation, epigenetic variation - such as differential DNA methylation - may exist between natural populations living under different environmental conditions. The goal of this paper was to test for morphological, genetic, and epigenetic differences between urban and rural populations within each of two species of Darwin’s finches. We found that *G. fortis* individuals at the urban site (Academy Bay) were larger than those at the rural site (El Garrapatero). In contrast, *G. fuliginosa* individuals did not differ morphologically between the sites. We did not find genetic differentiation between populations of either species based on CNV comparisons. However, we did find epigenetic (DMR) differences between urban and rural populations of both species of finches.

We found urban *G. fortis* were larger in nearly all morphological measurements compared to rural *G. fortis* (Table 1), which may be due to increased food availability at the urban site. Previous work suggests that urbanization around Academy Bay has relaxed selection on finch beak size [35, 36]. Urbanization is associated with a shift in the distribution of beak size in *G. fortis*: beak size is strongly bimodal at the rural site, whereas bimodality has decreased at the urban site concurrently with human population growth [35]. Both studies propose that increased food availability at the urban site has altered the selective landscape for *G. fortis* [35, 36]. Beak size is highly heritable in *Geospiza* finches; e.g. mid-parent vs. mid-offspring values estimate heritability of beak depth in *G. fortis* to be 0.74 [61].

In contrast, *G. fuliginosa* showed no morphological differentiation between sites (Table 1). *Geospiza fortis* is phenotypically more variable than *G. fuliginosa* on Santa Cruz Island [61]. As a result, *G. fortis* may have undergone more rapid local adaptation than *G. fuliginosa*. Although *G. fuliginosa* and *G. fortis* have overlapping dietary niches, they do show some degree of specialization [27]. It is possible that urbanization has had a greater selective effect on *G. fortis* than *G. fuliginosa*. Alternatively, morphological differences in *G. fortis* may be driven by hybridization between *G. fortis* and the slightly larger *G. magnirostris*. Hybridization between *G. fortis* and *G. magnirostris* has been documented on Santa Cruz [62]. While we have no information on relative rates of hybridization at our study sites, *G. magnirostris* is more abundant at the urban site than the rural site (4.56% of urban birds captured, compared to 1.86% of rural birds captured; unpublished data 2008–2016).

Despite differences in morphology between populations of *G. fortis*, we found no genetic differences between the urban and rural populations, based on the CNV comparisons made. Because CNV sequence coverage was limited, we may have overlooked small CNV, but larger CNV should have been detected between the two populations. CNV is a sensitive index of genetic differentiation between populations; indeed, some studies have found that CNV accounts for more genetic variation than SNPs [63–65]. Recent work has also linked CNV to rapid evolution in pepper moths [66] and primates [67].

Our study is first to explore genetic variation between populations of Darwin’s finches using large–scale genomic features (CNV). Like our study, previous studies using smaller-scale genomic markers (microsatellites, nuclear introns, and mitochondrial DNA) detected little or no genetic structure within populations of either *G. fortis* or *G. fuliginosa* [31, 34, 68]. Two recent studies of genomic variation among Darwin’s finches using SNPs did identify variable sites associated with variation in beak morphology [29, 30]. However, most of the genes associated with beak morphology in the two studies were different. These inconsistent results suggest that other forms of variation, such as large scale CNVs, could underlie phenotypic differences. However, our results show that negligible large size CNV changes exist between the rural and urban populations of *G. fortis* or *G. fuliginosa*.

In contrast to our genetic results, we found a large number of epigenetic differences between urban and rural populations in both species of finches and both cell types (Fig. 2). Although DMRs were found in both species, few of the same genomic regions were differentially methylated in *G. fortis* and *G. fuliginosa*. These data suggest that methylation patterns are species-specific, even when comparing closely related species. This may mean that *G. fortis* and *G. fuliginosa* are responding to environmental changes at the urban site in different ways. The lack of overlap in DMRs between the two species may reflect differences in their diets [27]. As discussed above, dietary differences may also have contributed to the morphological differences between urban and rural populations of *G. fortis.*

Although DMRs were also found in both cell types, few of the same genomic regions were differentially methylated in sperm and erythrocytes. Because methylation is involved with cell differentiation [6, 69], some lack of similarity in erythrocyte and sperm DMR is expected. The differences between the genomic regions that were differentially methylated in sperm and erythrocytes may provide clues as to the functional significance of the epimutations. DMRs in somatic cells, such as erythrocytes, potentially reflect effects of the environment on physiology of the birds. DMRs in germ cells, such as sperm, are more likely to be transgenerationally inherited and contribute to evolution. Recent studies show that heritability of methylation variants can be high, but that this varies among loci [12]. However, without following multiple generations of individuals with known ancestry, we cannot determine which of the DMRs in our study are heritable. It is possible that many of the DMRs we detected were plastic responses to the environment. Analysis of Darwin’s finches with known pedigrees - from long-term studies of banded populations - may be a way in which to distinguish heritable from non-heritable epimutations in the future.

While locations of DMRs varied between species and cell types (Fig. 4), they had genomic features in common. DMRs were typically 500–1000 bp in length (Fig. 3) and many were clustered in 2–7 Mb regions. Most DMRs were in areas of low CpG density known as “CpG deserts” (Fig. 5). Many studies of DNA methylation have focused on the gene-silencing effects of methylation in high-density “CpG islands” near transcriptional start sites [6]. However, DMRs in other genomic regions, such as CpG deserts, can have other important effects on gene regulation and expression [6, 70]. Methylation of cytosines increases the rate of cytosine to thymine transitions [71]. Thus, over time, methylation can cause CpG-poor regions in the genome to accumulate. The persistence of conserved clusters of methylated CpG sites within CpG deserts suggests that these regions are likely conserved and under purifying selection [70]. Thus, these types of DMRs may have a functional role in regulating gene expression and could be subject to selection.

Many of the DMRs we detected were associated with metabolic and signaling genes (Fig. 6). Previous work has suggested that novel food sources at the urban site are changing the diet of finches [27]. While we did not quantify phenotypic traits related to metabolism, it is possible that DMRs associated with metabolic genes are associated with other physiological differences between the urban and rural populations.

We also found DMRs associated with genes in the bone morphogenic protein (BMP) / transferring growth factor beta (TGFß) pathway (Fig. 7). Expression of Bmp4 is related to beak shape in *Geospiza* finches [72]; however, it is unknown what factors regulate gene expression at this locus. We previously found that this pathway was differentially methylated among species of Darwin’s finches [18]. These data suggest that DNA methylation may play a role in regulating expression of genes in this pathway and therefore may influence finch morphology.

Our study compared just two populations - one rural and one urban – and therefore we cannot be certain that urbanization is the key environmental change influencing finch morphology and/or epigenetics in our study. Moreover, it is possible that differences between the two populations are the result of epigenetic drift, rather than differential selection. Some dispersal of *G. fortis* between the urban and rural populations has been documented through mark-recapture studies; but it is not very common (J. Raeymaekers pers. comm.). Low levels of gene flow between populations would preclude divergence of the rural and urban populations due to drift. However, much more work is needed to understand the basis of epigenetic variation and its relationship to phenotypic variation in populations of Darwin’s finches.

## Conclusions

We found epigenetic differences between adjacent populations of each of two species of Darwin’s finches. We do not know which of the DMRs are responses to environmental differences between the urban and rural sites, versus the result of random epigenetic drift. However, as the environmental differences between our sites are recent (<60 years) any methylation changes associated with urbanization have spread quickly. As in other recent studies [19, 20, 22], the functional relationship between environmental and epigenetic variation is not well understood. Nevertheless, these results are consistent with a potential role of epigenetic variation in rapid adaptation to changing environments. Future studies are needed to determine what effects DMR have on phenotypes, and to what extent these methylation patterns may play a role in evolution.

## Additional files

Additional file 1: Figure S1. Comparison of vegetative cover at the rural site (El Garrapatero) versus urban site (Puerto Ayora, Academy Bay) over the course of the study. Cover was dervied from Normalized Difference Vegetative Index (NDVI) values generated from satellite imagery (ORNL DAAC. 2008. MODIS Collection 5 Land Products Global Subsetting and Visualization Tool. ORNL DAAC, Oak Ridge, Tennessee, USA. Accessed May 08, 2017 http://dx.doi.org/10.3334/ORNLDAAC/1241). Values range from 0-1 with 1 reprensenting the highest vegetation cover. (PDF 850 kb)

Additional file 2: Figure S2. Copy number variation (CNV) between the rural and urban populations. (A) CNV analysis summary for the *G. fortis* erythrocytes showing read depth and alignment, and CNV numbers per pool with chromosomes containing CNV indicated, and no overlap between rural and urban pools indicated. (B) CNV analysis summary for the *G. fuliginosa* erythrocytes with Read Mapping Summary, overall CNV per pool and chromosome, and no overlapping CNV identified. (PDF 20 kb)

Additional file 3: Table S1. The number of DMR detected at single window and multiple window scales at increasing levels of significance. (PDF 61 kb)

Additional file 4: Table S2. Description of multiple-window DMR detected in *G. fortis* sperm **(A)** and erythrocytes **(B)**. Description includes DMR name, chromosome number, DMR start site, length in base pair (bp), number of multiple sites, minimum p-value, CpG number per sequence length, CpG density (CpG number / 100 bp) and DMR gene association. “NA” indicates DMR associated with a gene that did not align to the zebra finch reference genome. (PDF 126 kb)

Additional file 5: Table S3. Description of multiple-window DMR detected in *G. fuliginosa* sperm **(A)** and erythrocytes **(B)**. Description includes DMR name, chromosome number, DMR start site, length in base pair (bp), number of multiple sites, minimum p-value, CpG number per sequence length, CpG density (CpG number / 100 bp) and DMR gene association. “NA” indicates DMR associated with a gene that did not align to the zebra finch reference genome. (PDF 154 kb)

Additional file 6: Table S4. Description of DMR clusters detected in *G. fortis* sperm **(A)** and erythrocytes **(B)** and *G. fuliginosa* sperm **(C)** and erythrocytes **(D)**. Description includes DMR in cluster, chromosome number, cluster start site, cluster stop site, length in bp, and minimum *p*-value. (PDF 103 kb)

Additional file 7: Table S5. Gene associations with DMR detected in *G. fortis* sperm **(A)** and erythrocytes **(B)** and *G. fuliginosa* sperm **(C)** and erythrocytes **(D)**. Description includes DMR name, gene symbol, entrez gene identification, chromosome number, start position site, ensemble gene identification number, gene description and gene classification category. (PDF 225 kb)

Additional file 8: Figure S3. MAPK signaling pathway. Genes associated with DMR are listed and outlined in red in the pathway. (PDF 109 kb)

Additional file 9: Figure S4. Glycolysis metabolism pathway. Genes associated with DMR are listed and outlined in red in the pathway. (PDF 66 kb)

Additional file 10: Figure S5. DMRs identified in pairwise comparison of pools within populations: **(A)** *G. Fuliginosa* RBC urban analysis, **(B)** *G. fuliginosa*-RBC rural analysis, **(C)** *G. fortis* RBC urban analysis, and **(D)** *G. fortis* rural analysis. Numbers indicate DMRs between urban (U) or rural (R) individual pools (1-3). “Full analysis” are DMRs identified between urban and rural pools. DMRs identified in the full analysis were found independently of within-site variation. (PDF 98 kb)

## Abbreviations

BMP: bone morphogenic protein
CNV: copy number variation
DDT: dithiothreitol
DMR: differentially DNA methylated region
IP: immunoprecipitation
LMM: linear mixed effects models
NGS: next generation sequencing
PBS: Phosphate Buffered Saline
TGFß: transferring growth factor beta
