# Supplementary figures and images for: Epigenetic variation between urban and rural populations of Darwin’s finches
Source: BMC Evol Biol. 2017 Aug 24;17:183. doi: 10.1186/s12862-017-1025-9 (PMC5569522; doi:10.1186/s12862-017-1025-9)

Supplemental Figure S1.

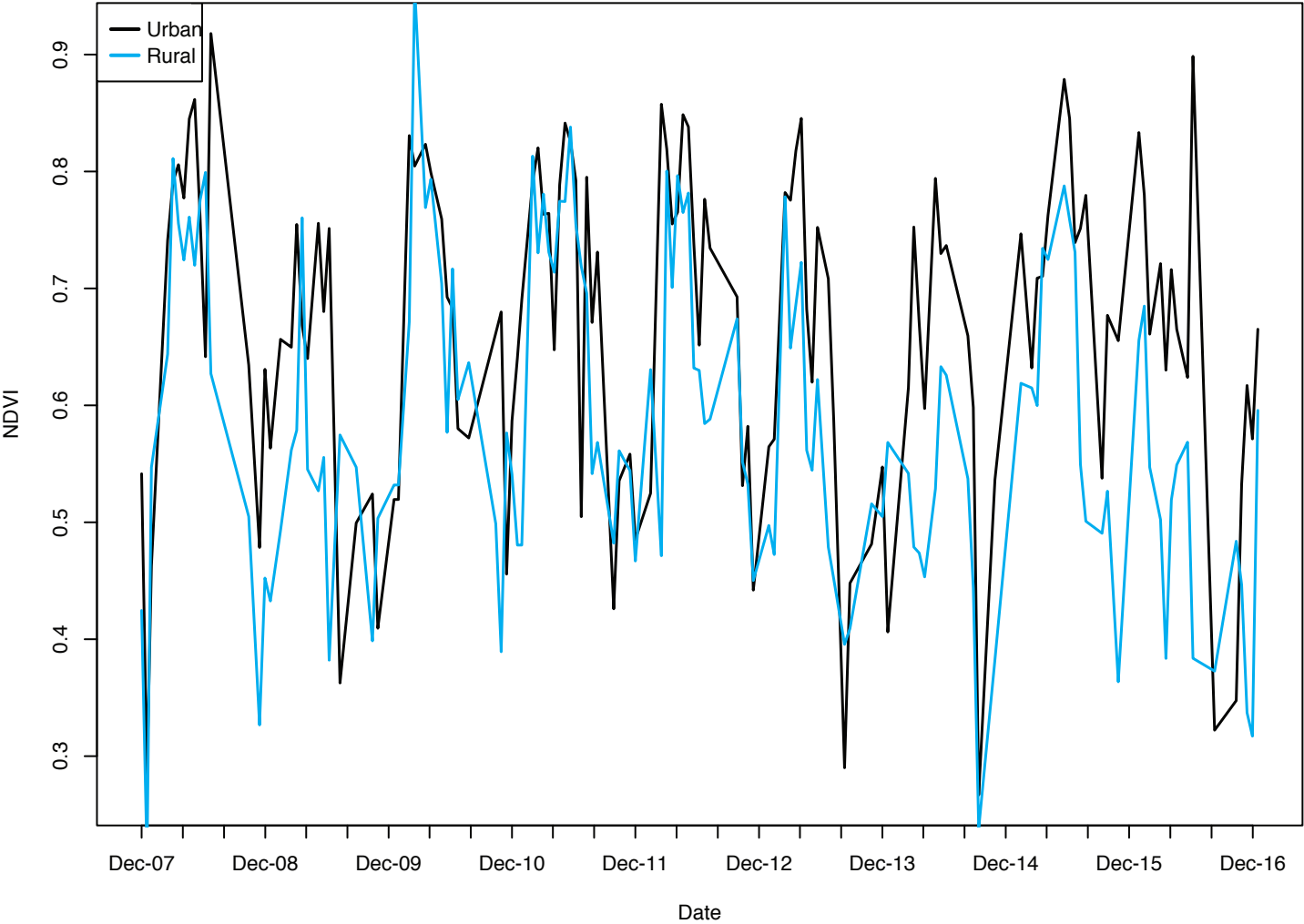

Supplement: Supplementary file 1 — Comparison of vegetative cover at the rural site (El Garrapatero) versus urban site (Puerto Ayora, Academy Bay) over the course of the study. Cover was dervied from Normalized Difference Vegetative Index (NDVI) values generated from satellite imagery (ORNL DAAC. 2008. MODIS Collection 5 Land Products Global Subsetting and Visualization Tool. ORNL DAAC, Oak Ridge, Tennessee, USA. Accessed May 08, 2017 http://dx.doi.org/10.3334/ORNLDAAC/1241). Values range from 0-1 with 1 reprensenting the highest vegetation cover. (PDF 850 kb) [file 12862_2017_1025_MOESM1_ESM.pdf]

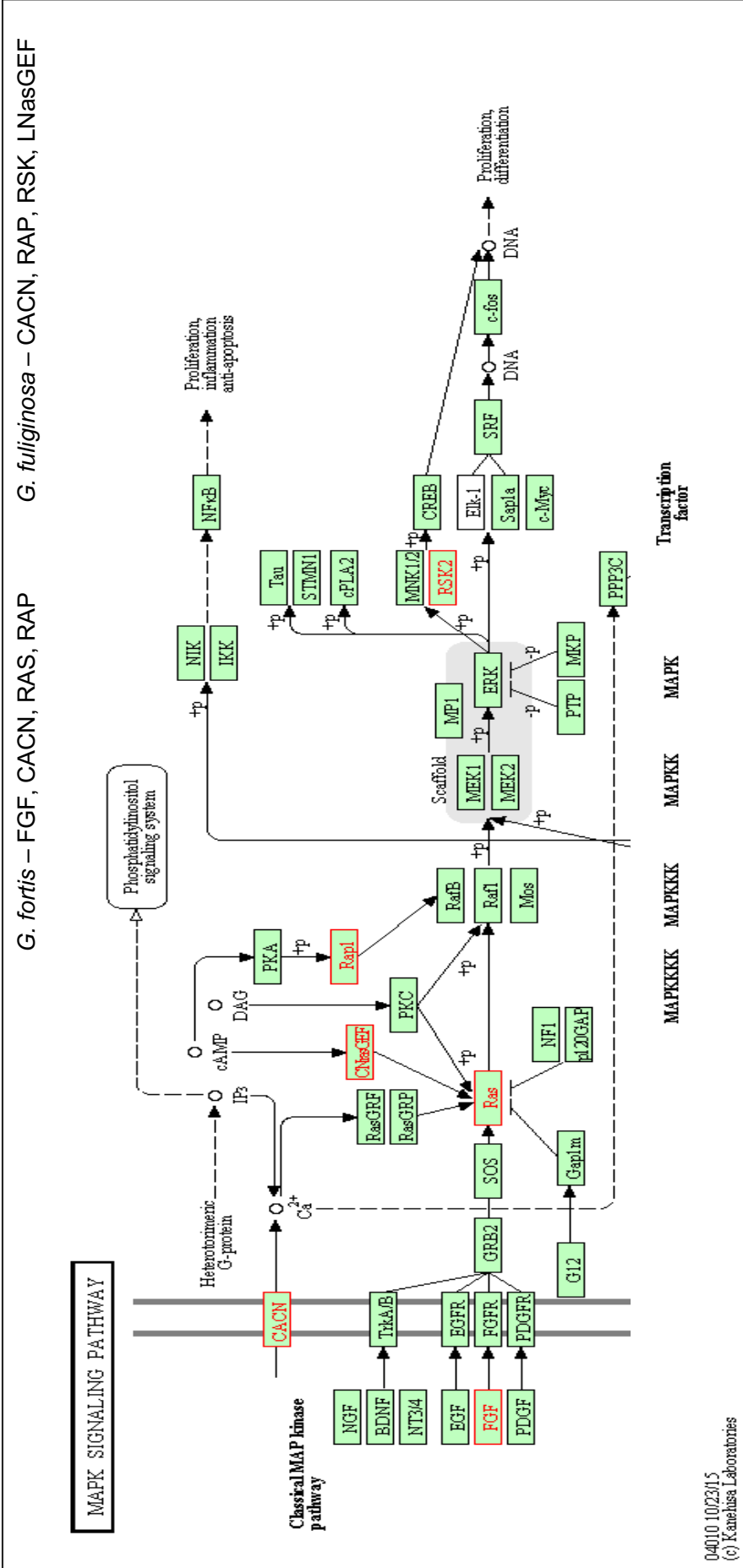

Supplement: Supplementary file 8 — MAPK signaling pathway. Genes associated with DMR are listed and outlined in red in the pathway. (PDF 109 kb) [file 12862_2017_1025_MOESM8_ESM.pdf]

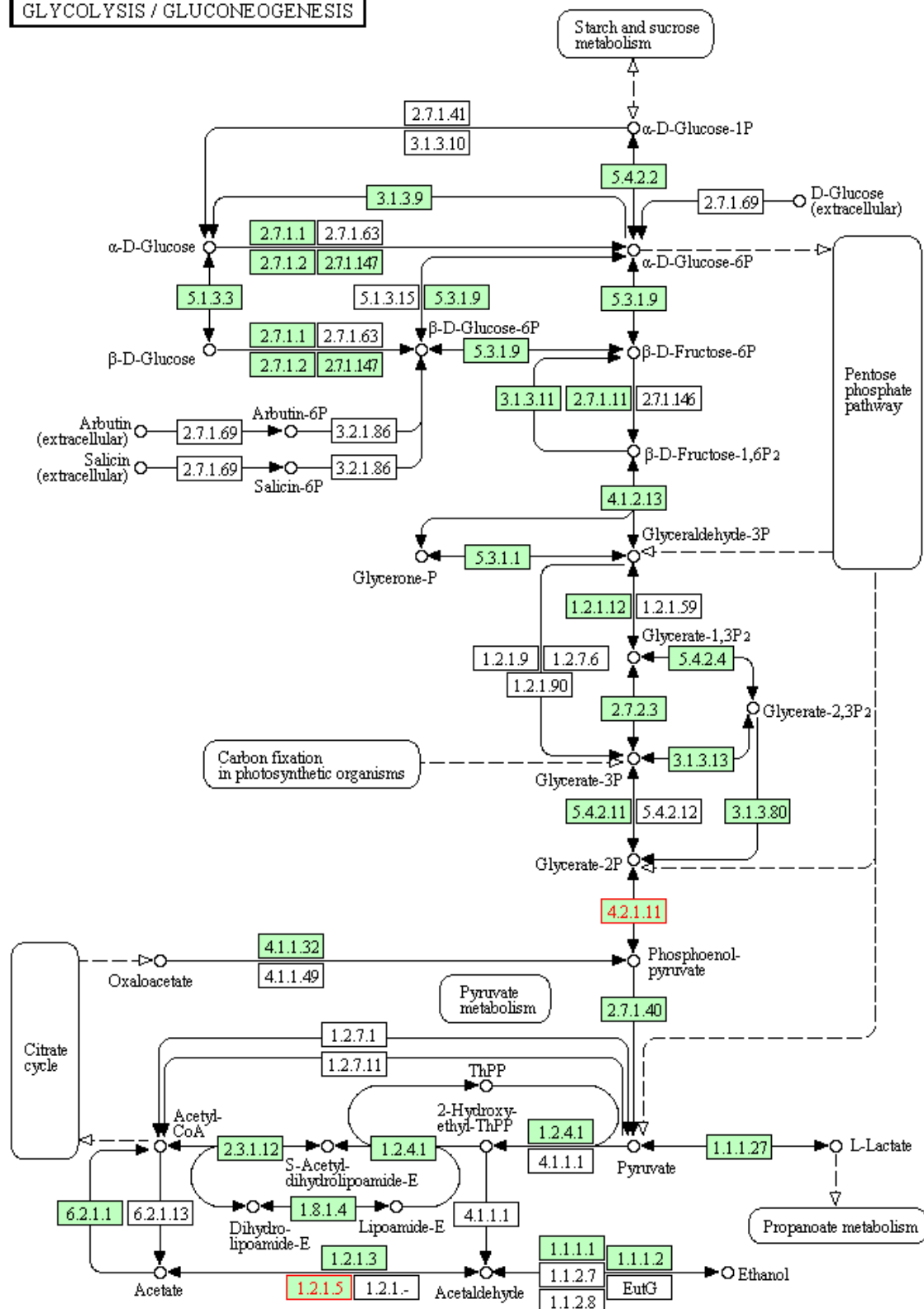

Supplement: Supplementary file 9 — Glycolysis metabolism pathway. Genes associated with DMR are listed and outlined in red in the pathway. (PDF 66 kb) [file 12862_2017_1025_MOESM9_ESM.pdf]
