# Supplementary material for: Epigenetic variation between urban and rural populations of Darwin’s finches
Source: BMC Evol Biol. 2017 Aug 24;17:183. doi: 10.1186/s12862-017-1025-9 (PMC5569522; doi:10.1186/s12862-017-1025-9)
Supplement: Supplementary file 2 — Copy number variation (CNV) between the rural and urban populations. (A) CNV analysis summary for the G. fortis erythrocytes showing read depth and alignment, and CNV numbers per pool with chromosomes containing CNV indicated, and no overlap between rural and urban pools indicated. (B) CNV analysis summary for the G. fuliginosa erythrocytes with Read Mapping Summary, overall CNV per pool and chromosome, and no overlapping CNV identified. (PDF 20 kb) [file 12862_2017_1025_MOESM2_ESM.pdf]

Supplemental Figure S2

CNV Analysis of Individual Sample Pools

(A) CNV analysis summary for the *G.fortis* RBC (erythrocytes)

Read Mapping Summary:

	FB1	FB2	FB3	FB4	FB5	FB6
Read Number	31943702	35390572	35292876	32866339	32098287	33303537
Overall Alignment Rate	48.12%	48.85%	48.30%	48.10%	48.44%	47.99%

The number of reads present for each sample and the overall alignment rate calculated by bowtie2.

Overall CNV numbers per pool and chromosome

	18	7	27	3	9	Un	11	Z
**sFB1.bam**	1	1	0	0	0	0	0	0
**sFB2.bam**	0	1	1	1	1	1	0	0
**sFB6.bam**	0	0	0	0	0	0	1	1

The number of CNV found and separated by sample and chromosome

Overlapping CNVs between rural and urban populations

There were no CNV called in all rural pools or all urban pools.

(B) CNV analysis summary for the *G. fuliginosa* RBC

Read Mapping Summary:

	FB1	FB2	FB3	FB4	FB5	FB6
Read Number	29702409	35909278	32460939	31290666	31033219	39220844
Overall Alignment Rate	44.77%	47.91%	48.62%	48.11%	46.76%	45.51%

The number of reads present for each sample and the overall alignment rate calculated by bowtie2.

Overall CNV numbers per pool and chromosome

	4	20	1	10	11	13	14	18	1A	2	3	5	6	7	8	9	Un	Z
**sFB10.bam**	1	0	0	0	0	0	0	0	0	0	0	0	0	0	0	0	0	0
**sFB11.bam**	0	1	0	0	0	0	0	0	0	0	0	0	0	0	0	0	0	0
**sFB12.bam**	32	0	51	4	8	1	3	1	34	41	34	29	13	18	16	6	21	20
**sFB8.bam**	0	0	0	0	0	0	0	0	0	1	0	0	0	0	0	0	0	0
**sFB9.bam**	0	1	0	0	0	0	0	0	0	0	0	0	0	0	0	0	0	0

The number of CNV found and separated by sample and chromosome

Overlapping CNVs between rural and urban populations

There were no CNV called in all rural or all urban pools exclusively.
