# Supplementary material for: Epigenetic variation between urban and rural populations of Darwin’s finches
Source: BMC Evol Biol. 2017 Aug 24;17:183. doi: 10.1186/s12862-017-1025-9 (PMC5569522; doi:10.1186/s12862-017-1025-9)
Supplement: Supplementary file 3 — The number of DMR detected at single window and multiple window scales at increasing levels of significance. (PDF 61 kb) [file 12862_2017_1025_MOESM3_ESM.pdf]

Table S1. The number of DMR detected at single window and multiple window scales at increasing levels of significance.

A. *G. fortis* Sperm

Number of DMRs vs. p-value cutoff

**p-value**	**Single Window**	**Multiple Windows**
0.01	24,058	44,423
0.001	1270	110
1e-04	79	2
1e-05	5	0

B. *G. fuliginosa* Sperm

Number of DMRs vs. p-value cutoff

**p-value**	**Single Window**	**Multiple Windows**
0.01	42,495	8,800
0.001	1,904	139
1e-04	22	1
1e-05	1	0

C. *G. fortis* Erythrocytes

Number of DMRs vs. p-value cutoff

**p-value**	**Single Window**	**Multiple Windows**
0.01	27,127	3,087
0.001	2,871	129
1e-04	252	4
1e-05	20	0

D. *G. fuliginosa* Erythrocytes

Number of DMRs vs. p-value cutoff

**p-value**	**Single Window**	**Multiple Windows**
0.01	35,541	5,432
0.001	4,663	324
1e-04	493	11
1e-05	66	3
