# Supplementary material for: Epigenetic variation between urban and rural populations of Darwin’s finches
Source: BMC Evol Biol. 2017 Aug 24;17:183. doi: 10.1186/s12862-017-1025-9 (PMC5569522; doi:10.1186/s12862-017-1025-9)
Supplement: Supplementary file 4 — Description of multiple-window DMR detected in G. fortis sperm (A) and erythrocytes (B). Description includes DMR name, chromosome number, DMR start site, length in base pair (bp), number of multiple sites, minimum p-value, CpG number per sequence length, CpG density (CpG number / 100 bp) and DMR gene association. “NA” indicates DMR associated with a gene that did not align to the zebra finch reference genome. (PDF 126 kb) [file 12862_2017_1025_MOESM4_ESM.pdf]

Supplemental Table S2A *G. fortis* **Multiple-Window DMR Sperm List**

DMR Name	Chr	Start	Length (bp)	# Sites	min P Value	CpG #	CpG Density (#/100bp)	Gene Association
DMR1:2405001	1	2405001	1400	2	1.88E-05	1	0.07	
DMR1:17460701	1	17460701	2200	2	0.00083337	4	0.1	
DMR1:89900301	1	89900301	300	2	0.00024454	10	3.3	ENO2;LRRC23
DMR1A:31501	1A	31501	200	2	0.00056486	0	0	
DMR1A:9608801	1A	9608801	400	2	0.00039257	10	2.5	HGF
DMR1A:33384201	1A	33384201	400	2	0.00042233	1	0.2	MSRB3
DMR2:1016201	2	1016201	3000	2	0.00017649	56	1.8	CCDC12
DMR2:1382701	2	1382701	4200	2	6.06E-05	32	0.7	
DMR2:11077401	2	11077401	500	2	7.82E-05	16	3.2	NA
DMR2:49493301	2	49493301	800	2	0.00017788	8	1	
DMR2:52702101	2	52702101	2600	2	0.00047911	115	4.4	
DMR2:57021501	2	57021501	300	2	0.00055811	3	1	
DMR2:57355101	2	57355101	1100	2	0.00043181	24	2.1	RPL15
DMR2:105076801	2	105076801	2000	2	3.16E-05	19	0.9	
DMR2:152511201	2	152511201	3100	2	0.00032751	115	3.7	NA
DMR2:155551401	2	155551401	1000	2	0.00018367	24	2.4	RHPN1
DMR3:19616201	3	19616201	200	2	0.00018836	2	1	GALNT14
DMR3:22144501	3	22144501	500	2	9.00E-05	1	0.2	NRXN1
DMR3:82421001	3	82421001	1200	2	8.39E-05	80	6.6	HTR1B
DMR3:110673201	3	110673201	1600	2	0.00013009	31	1.9	PAQR8
DMR3:111805401	3	111805401	200	2	7.92E-05	2	1	MSRA
DMR3:112515501	3	112515501	900	2	0.00018417	47	5.2	FZD3
DMR4:5948301	4	5948301	300	2	0.00012392	5	1.6	
DMR4:6462901	4	6462901	2700	2	0.00050397	111	4.1	FAT4
DMR4:10513201	4	10513201	1000	3	0.00034909	63	6.3	LRBA
DMR4:58976401	4	58976401	500	2	3.21E-05	26	5.2	JAKMIP1
DMR4:67704101	4	67704101	800	2	4.79E-05	56	7	CTNNA2;LRRTM1
DMR4A:4743001	4A	4743001	400	2	0.00050104	1	0.2	RPS6KA6
DMR4A:15730101	4A	15730101	800	3	0.0003688	20	2.5	
DMR4A:17926101	4A	17926101	1200	2	0.00036737	12	1	
DMR4A:19342701	4A	19342701	1400	2	0.00048117	44	3.1	CNGA2
DMR5:6667801	5	6667801	9500	3	0.00032085	378	3.9	TMEM132A;CD6
DMR5:10950801	5	10950801	2000	2	7.05E-05	17	0.8	SOX6
DMR5:14025601	5	14025601	2000	2	9.59E-05	19	0.9	SYT8
DMR5:43833301	5	43833301	1100	2	0.0004157	30	2.7	
DMR5:58425401	5	58425401	1000	2	0.00024649	15	1.5	
DMR6:3861701	6	3861701	900	2	0.00024665	55	6.1	SGMS1
DMR6:21155001	6	21155001	1400	2	0.00036418	37	2.6	NA
DMR7:1620001	7	1620001	1100	2	0.00045746	25	2.2	HDAC4
DMR7:2068201	7	2068201	4000	4	0.00011985	55	1.3	
DMR8:24996701	8	24996701	500	2	0.00031729	15	3	NFIA
DMR9:3092101	9	3092101	2700	2	0.00018478	19	0.7	
DMR9:16765501	9	16765501	500	2	0.00028966	14	2.8	
DMR9:20816401	9	20816401	1200	2	0.00045795	1	0.08	
DMR9:23196601	9	23196601	500	2	0.00089336	24	4.8	NA
DMR10:2121101	10	2121101	10600	2	0.00027567	542	5.1	PLEC
DMR10:14143801	10	14143801	2500	3	0.00038843	78	3.1	NA
DMR10:14466301	10	14466301	200	2	2.98E-05	10	5	SV2B
DMR10:15617101	10	15617101	500	2	0.0008557	17	3.4	
DMR10:17182001	10	17182001	2800	2	2.67E-05	61	2.1	IGF1R
DMR11:2277401	11	2277401	600	2	8.54E-05	25	4.1	
DMR11:14550401	11	14550401	400	2	0.00015349	4	1	
DMR11:16380101	11	16380101	800	2	0.00052102	7	0.8	NA
DMR12:1610001	12	1610001	800	2	5.31E-05	3	0.3	
DMR12:4785401	12	4785401	2400	2	0.00014665	119	4.9	MST1R

DMR12:5901001	12	5901001	1100	2	0.00029715	23	2	NA
DMR13:11920001	13	11920001	300	2	0.00067146	22	7.3	GABRB2
DMR14:1181901	14	1181901	300	3	8.90E-05	20	6.6	RAB40C
DMR14:3090701	14	3090701	1300	2	0.00065447	28	2.1	RHBDF1
DMR14:8978801	14	8978801	1100	2	0.00038178	37	3.3	
DMR14:15047701	14	15047701	1300	2	0.00039071	0	0	
DMR15:2627801	15	2627801	200	2	1.97E-05	7	3.5	NA
DMR15:2915401	15	2915401	200	2	0.00019932	9	4.5	IFT81
DMR15:5142701	15	5142701	800	2	0.00032215	2	0.2	
DMR17:410701	17	410701	3000	2	0.00021266	80	2.6	
DMR17:3431501	17	3431501	700	2	4.00E-05	34	4.8	NA
DMR18:991001	18	991001	2200	2	2.96E-06	144	6.5	BTBD17
DMR18:1081001	18	1081001	1100	2	0.00044212	40	3.6	SLC38A10
DMR18:1084101	18	1084101	6900	2	0.00040113	216	3.1	SLC38A10
DMR19:514001	19	514001	1000	2	0.00048372	36	3.6	RNF43
DMR19:5685501	19	5685501	300	2	0.00010526	11	3.6	RFFL
DMR20:353601	20	353601	1000	2	0.00011803	16	1.6	
DMR20:553601	20	553601	1700	2	2.41E-05	19	1.1	RALGAPB
DMR20:1139801	20	1139801	300	2	3.27E-05	10	3.3	NA
DMR20:12107601	20	12107601	1100	2	0.00037116	2	0.1	
DMR20:13641501	20	13641501	300	2	0.00010363	15	5	NA
DMR21:3356301	21	3356301	4000	2	0.00035826	87	2.1	PLCH2
DMR23:4735301	23	4735301	1500	2	0.00070343	47	3.1	EPHA10
DMR25:797501	25	797501	1500	2	0.00080997	59	3.9	NA
DMR26:2781201	26	2781201	800	2	0.00034282	14	1.7	
DMR27:383501	27	383501	2100	2	7.14E-06	108	5.1	DDX42
DMR27:4437801	27	4437801	7000	4	0.00019975	211	3	NA
DMR28:3706001	28	3706001	300	2	0.00051934	0	0	
DMR28:4853401	28	4853401	2900	3	0.00043561	170	5.8	KLHL26
DMRZ:528401	Z	528401	600	2	0.00020996	28	4.6	
DMRZ:34124901	Z	34124901	300	2	1.80E-05	0	0	
DMRZ:38519001	Z	38519001	200	2	0.000281	7	3.5	RPP25
DMRUn:1762101	Un	1762101	300	2	0.00091328	1	0.3	
DMRUn:13189701	Un	13189701	400	2	4.06E-05	2	0.5	
DMRUn:37518901	Un	37518901	200	2	0.00010471	15	7.5	
DMRUn:38440401	Un	38440401	1800	2	0.0006965	112	6.2	ARHGAP39
DMRUn:42636001	Un	42636001	200	2	0.00035246	4	2	
DMRUn:52965301	Un	52965301	1000	3	7.17E-05	3	0.3	
DMRUn:64017401	Un	64017401	1000	2	0.00024876	9	0.9	
DMRUn:65244001	Un	65244001	200	2	5.10E-05	12	6	
DMRUn:70786601	Un	70786601	11700	5	0.00015993	25	0.2	SYCP1
DMRUn:70799501	Un	70799501	3000	3	0.00012449	23	0.7	SYCP1
DMRUn:78679901	Un	78679901	300	2	0.00051026	3	1	
DMRUn:93117201	Un	93117201	700	2	6.42E-05	1	0.1	
DMRUn:93590701	Un	93590701	1200	2	0.00018951	4	0.3	
DMRUn:116431501	Un	116431501	2000	2	0.00050843	79	3.9	DNMT1
DMRUn:116729701	Un	116729701	1400	2	0.00079537	14	1	DOCK7
DMRUn:124049301	Un	124049301	300	2	3.29E-05	1	0.3	
DMRUn:132241501	Un	132241501	2200	2	0.00067437	86	3.9	USP21
DMRUn:134552701	Un	134552701	1500	2	0.00012924	7	0.4	
DMRUn:142781801	Un	142781801	200	2	0.00017126	13	6.5	
DMRUn:144276901	Un	144276901	700	4	2.93E-05	5	0.7	
DMRUn:155480601	Un	155480601	400	2	0.00036774	1	0.2	
DMRUn:156914801	Un	156914801	2200	3	0.00021333	128	5.8	
DMRUn:173298101	Un	173298101	300	2	0.00040971	20	6.6	

Supplemental Table S2B ***G. fortis*** **Multiple-Window DMR Erythrocyte List**

DMR Name	Chr	Start	Length (bp)	# Sites	min P Value	CpG #	CpG Density (#/100bp)	Gene Association
DMR1:2682401	1	2682401	900	2	0.00027256	0	0	NA
DMR1:5421101	1	5421101	200	2	1.02E-06	2	1	CXorf36
DMR1:15978201	1	15978201	1100	2	3.20E-05	7	0.6	
DMR1:17714201	1	17714201	600	2	4.03E-05	3	0.5	GEMIN8
DMR1:26248601	1	26248601	300	2	0.00014309	6	2	
DMR1:30831101	1	30831101	200	2	0.00018354	1	0.5	
DMR1:34061101	1	34061101	1200	2	4.96E-05	2	0.1	
DMR1:48999201	1	48999201	1300	2	1.53E-05	6	0.4	
DMR1:52621901	1	52621901	400	2	0.00030553	5	1.2	NA
DMR1:59413201	1	59413201	200	2	0.00022223	1	0.5	NA
DMR1:68474101	1	68474101	300	2	0.00045777	0	0	PIBF1
DMR1:83012401	1	83012401	300	2	0.00041654	1	0.3	NA
DMR1:93094801	1	93094801	300	2	0.00019656	1	0.3	NA
DMR1:93731001	1	93731001	200	2	0.00013012	0	0	
DMR1:98009701	1	98009701	1700	2	0.00042053	9	0.5	
DMR1:103888201	1	103888201	700	2	0.00037939	5	0.7	
DMR1:116098201	1	116098201	300	2	8.32E-05	1	0.3	
DMR1:117036401	1	117036401	200	2	0.00020596	1	0.5	
DMR1A:14851501	1A	14851501	1200	2	0.00028406	5	0.47	CPNE8
DMR1A:33667601	1A	33667601	200	2	0.00014771	0	0	
DMR1A:37330801	1A	37330801	200	2	0.00050789	0	0	
DMR1A:37641201	1A	37641201	800	3	7.67E-06	9	1.1	
DMR1A:46028501	1A	46028501	500	2	4.03E-05	6	1.2	IKBIP
DMR1A:47095601	1A	47095601	2200	2	0.0002731	13	0.5	NA
DMR1A:51403801	1A	51403801	400	2	4.03E-05	1	0.2	
DMR1A:68987201	1A	68987201	300	2	3.61E-05	1	0.3	
DMR1A:70257101	1A	70257101	700	2	4.78E-05	6	0.8	MICAL3
DMR2:1798801	2	1798801	1100	2	0.00015174	26	2.3	
DMR2:6567801	2	6567801	200	2	7.19E-05	0	0	
DMR2:25864201	2	25864201	300	2	0.00022459	4	1.3	
DMR2:44270401	2	44270401	200	2	0.00053621	3	1.5	FARS2
DMR2:48865301	2	48865301	200	2	0.00040403	0	0	
DMR2:74709601	2	74709601	200	2	0.00012255	4	2	
DMR2:81217201	2	81217201	200	2	0.00020906	0	0	
DMR2:106386901	2	106386901	1700	2	0.00072119	15	0.8	
DMR2:106836801	2	106836801	1200	2	0.00067838	11	0.9	
DMR2:109922901	2	109922901	1300	2	0.00024475	8	0.6	
DMR2:127963201	2	127963201	200	2	0.00027855	1	0.5	
DMR2:135732501	2	135732501	800	2	6.12E-05	6	0.7	
DMR2:139628601	2	139628601	800	2	0.00094242	4	0.5	
DMR2:148037901	2	148037901	800	3	2.14E-05	3	0.3	NA
DMR2:153195201	2	153195201	1300	2	0.00039388	9	0.6	
DMR3:16726301	3	16726301	2600	2	0.00049046	14	0.5	
DMR3:17261701	3	17261701	200	2	1.87E-06	1	0.5	
DMR3:24857301	3	24857301	200	2	0.00027559	1	0.5	
DMR3:26735901	3	26735901	200	2	7.67E-06	1	0.5	
DMR3:31219401	3	31219401	200	2	0.00040117	4	2	
DMR3:35391201	3	35391201	500	2	4.11E-05	1	0.2	CRIM1
DMR3:78517101	3	78517101	300	2	1.11E-06	3	1	

DMR3:78536501	3	78536501	1300	2	0.00027855	4	0.3	
DMR3:91910801	3	91910801	400	2	3.96E-05	2	0.5	
DMR3:104372501	3	104372501	200	2	0.00011622	1	0.5	
DMR4:34475501	4	34475501	900	2	0.00061095	4	0.4	
DMR4:50910901	4	50910901	900	2	0.00012234	52	5.7	
DMR4:61178101	4	61178101	300	2	3.99E-05	0	0	
DMR4A:6107001	4A	6107001	200	2	0.0004287	3	1.5	
DMR5:2675901	5	2675901	1400	2	7.94E-05	12	0.8	
DMR5:2884401	5	2884401	200	2	0.00025439	0	0	ELP4
DMR5:11814401	5	11814401	200	2	7.66E-05	2	1	
DMR5:14504501	5	14504501	1200	2	1.05E-05	7	0.5	
DMR5:17085201	5	17085201	1100	2	0.00018656	6	0.5	NA
DMR5:25257301	5	25257301	700	2	8.59E-06	35	5	
DMR5:27884501	5	27884501	200	2	5.89E-05	0	0	
DMR5:38477401	5	38477401	200	2	5.06E-05	4	2	
DMR5:47907301	5	47907301	400	2	1.10E-05	1	0.2	
DMR6:4069801	6	4069801	200	2	1.96E-05	2	1	NA
DMR6:10248601	6	10248601	200	2	0.00024475	0	0	
DMR6:15841201	6	15841201	200	2	0.00016098	1	0.5	GBF1
DMR6:22127301	6	22127301	1500	2	4.03E-05	4	0.2	FBXW4;FGF8
DMR6:24244501	6	24244501	200	2	0.00052526	2	1	SORCS3
DMR6:24443901	6	24443901	200	2	0.00022223	1	0.5	
DMR7:4454501	7	4454501	200	2	0.00056941	0	0	
DMR7:7103301	7	7103301	200	2	0.00048932	3	1.5	MRAS
DMR7:7578001	7	7578001	200	2	0.00024475	0	0	
DMR7:22822301	7	22822301	2700	2	2.60E-05	6	0.2	
DMR7:37235801	7	37235801	1800	2	0.00023034	16	0.8	
DMR7:37271201	7	37271201	200	2	0.00014816	2	1	
DMR7:38670601	7	38670601	2100	2	0.00023782	25	1.1	
DMR8:1651101	8	1651101	200	2	6.12E-05	1	0.5	
DMR8:9281501	8	9281501	300	2	0.00034517	3	1	PTBP2
DMR9:19271201	9	19271201	1500	2	0.00022223	9	0.6	
DMR10:11658001	10	11658001	300	2	4.96E-05	3	1	
DMR10:11904101	10	11904101	1000	2	0.00054934	9	0.9	
DMR10:14265201	10	14265201	800	3	0.00033345	2	0.2	
DMR10:18696001	10	18696001	300	2	0.00039166	1	0.3	
DMR11:7908101	11	7908101	300	2	0.00012243	1	0.3	
DMR12:7343801	12	7343801	900	2	0.00048932	4	0.4	
DMR12:9477701	12	9477701	400	2	9.44E-05	1	0.2	
DMR12:10036401	12	10036401	600	2	4.39E-05	8	1.3	ABTB1
DMR12:11939801	12	11939801	200	2	0.00070523	7	3.5	
DMR12:20282601	12	20282601	300	2	0.00060805	2	0.6	ITPR1
DMR13:246201	13	246201	300	2	0.00020988	0	0	STK32A
DMR13:11691501	13	11691501	900	2	0.00026847	5	0.5	NA
DMR14:781701	14	781701	200	2	0.00034848	1	0.5	
DMR14:8553401	14	8553401	700	2	0.00056809	6	0.8	
DMR14:10140501	14	10140501	200	2	0.00042141	1	0.5	CACNA1H;B9D1
DMR15:5515601	15	5515601	200	2	0.00014552	1	0.5	MN1
DMR15:9613601	15	9613601	300	2	0.00024475	1	0.3	
DMR15:12868101	15	12868101	200	2	0.0002835	1	0.5	NA
DMR15:13976501	15	13976501	400	2	4.04E-06	3	0.7	
DMR15:14137901	15	14137901	900	2	1.20E-05	5	0.5	AIFM3

DMR17:7618801	17	7618801	300	2	0.00021721	3	1	
DMR18:7751401	18	7751401	200	2	0.0001428	5	2.5	ST6GALNAC2
DMR18:10818401	18	10818401	500	2	5.76E-05	2	0.4	
DMR19:6307401	19	6307401	200	2	0.00015843	3	1.5	
DMR19:9135801	19	9135801	300	2	5.29E-05	3	1	NA
DMR20:4414401	20	4414401	300	3	3.52E-05	4	1.3	
DMR20:5555701	20	5555701	300	2	4.22E-05	5	1.6	
DMR20:6871001	20	6871001	1500	2	0.00065159	7	0.4	
DMR20:10911801	20	10911801	1600	2	0.00067837	7	0.4	
DMR20:15138501	20	15138501	1400	2	4.46E-05	21	1.5	KCNB1
DMR21:4415901	21	4415901	200	2	0.00041562	0	0	ACAP3
DMR26:3225201	26	3225201	300	2	1.55E-05	3	1	RAP1A
DMR26:4154601	26	4154601	400	2	0.00064105	5	1.2	
DMRZ:25875601	Z	25875601	200	2	0.00097829	0	0	
DMRZ:32978101	Z	32978101	900	2	0.00038839	5	0.5	
DMRZ:45594501	Z	45594501	200	2	6.12E-05	0	0	
DMRZ:45691301	Z	45691301	300	2	0.00030731	4	1.3	ISL1
DMRZ:70647701	Z	70647701	1200	2	2.69E-05	0	0	EDIL3
DMRUn:31643901	Un	31643901	900	2	0.00012679	1	0.1	
DMRUn:38098901	Un	38098901	200	2	0.00014552	1	0.5	
DMRUn:39304701	Un	39304701	200	2	6.12E-05	1	0.5	
DMRUn:48843301	Un	48843301	300	2	6.12E-05	1	0.3	
DMRUn:73071501	Un	73071501	400	2	1.06E-05	4	1	
DMRUn:81523201	Un	81523201	1000	2	0.00063847	14	1.4	
DMRUn:115914401	Un	115914401	200	2	0.00097829	0	0	NA
DMRUn:130440301	Un	130440301	200	2	0.00073111	2	1	
DMRUn:166943301	Un	166943301	800	2	1.35E-05	4	0.5	
DMRUn:167343801	Un	167343801	300	2	3.12E-05	3	1	NA
