# Supplementary material for: Epigenetic variation between urban and rural populations of Darwin’s finches
Source: BMC Evol Biol. 2017 Aug 24;17:183. doi: 10.1186/s12862-017-1025-9 (PMC5569522; doi:10.1186/s12862-017-1025-9)
Supplement: Supplementary file 5 — Description of multiple-window DMR detected in G. fuliginosa sperm (A) and erythrocytes (B). Description includes DMR name, chromosome number, DMR start site, length in base pair (bp), number of multiple sites, minimum p-value, CpG number per sequence length, CpG density (CpG number / 100 bp) and DMR gene association. “NA” indicates DMR associated with a gene that did not align to the zebra finch reference genome. (PDF 154 kb) [file 12862_2017_1025_MOESM5_ESM.pdf]

Supplemental Table S3A *G. fuliginosa* **Multiple-Window DMR Sperm List**

DMR Name	Chr	Start	Length (bp)	# Sites	min P Value	CpG #	CpG Density (#/100bp)	Gene Association
DMR1:10654201	1	10654201	1200	2	0.00028226	3	0.2	IL1RAPL1
DMR1:14148101	1	14148101	1300	2	0.00065919	1	0.07	
DMR1:25398601	1	25398601	1900	2	0.00056475	73	3.8	
DMR1:51009601	1	51009601	1800	2	0.00074605	4	0.2	
DMR1:57226101	1	57226101	2300	2	0.00059756	10	0.4	
DMR1:60559501	1	60559501	200	2	0.0008456	0	0	
DMR1:66164301	1	66164301	1600	2	3.79E-05	5	0.3	
DMR1:71703301	1	71703301	400	2	9.86E-05	1	0.2	
DMR1:80766701	1	80766701	300	2	0.00042544	1	0.3	FAT3
DMR1:89065301	1	89065301	1100	2	0.00010216	4	0.3	
DMR1:89740001	1	89740001	400	2	0.00026565	1	0.2	
DMR1:94245001	1	94245001	3100	2	3.25E-05	13	0.4	
DMR1:96225201	1	96225201	1400	2	0.00088771	3	0.2	
DMR1:110763501	1	110763501	700	2	0.00033142	1	0.1	
DMR1A:5308001	1A	5308001	2500	2	0.0004006	3	0.1	
DMR1A:6286301	1A	6286301	800	2	0.00012642	2	0.2	
DMR1A:11577701	1A	11577701	1100	2	0.00010458	2	0.1	PTPN12
DMR1A:13180201	1A	13180201	600	2	0.0005454	3	0.5	
DMR1A:22795501	1A	22795501	1300	2	7.56E-05	4	0.3	
DMR1A:24114101	1A	24114101	1600	2	0.00061683	101	6.3	WNT2
DMR1A:25971701	1A	25971701	900	2	0.00052497	1	0.1	
DMR1A:27064701	1A	27064701	500	2	0.0003543	1	0.2	IMMP2L
DMR1A:29521501	1A	29521501	600	2	0.00068445	0	0	TMEM117
DMR1A:33582601	1A	33582601	1000	2	0.00050694	2	0.2	
DMR1A:37122201	1A	37122201	1200	2	4.58E-05	5	0.4	NA
DMR1A:48389101	1A	48389101	3200	2	0.0001117	8	0.2	TTC26
DMR1A:59545101	1A	59545101	1100	2	0.00084561	3	0.2	KDM7A
DMR1A:61248301	1A	61248301	200	2	0.00041825	0	0	C2CD5
DMR2:7094701	2	7094701	600	2	0.00067655	0	0	GALNT11
DMR2:8947601	2	8947601	1100	2	0.00056393	4	0.3	
DMR2:19980701	2	19980701	2800	2	0.00056404	109	3.8	NA
DMR2:21027601	2	21027601	1800	2	0.00072248	6	0.3	
DMR2:21459501	2	21459501	1300	2	4.60E-05	4	0.3	NA
DMR2:22412201	2	22412201	1300	3	0.0001609	2	0.1	
DMR2:44117701	2	44117701	600	2	0.00017683	5	0.8	
DMR2:46614201	2	46614201	300	2	0.00078566	0	0	
DMR2:47305801	2	47305801	500	2	0.00022533	1	0.2	
DMR2:57223201	2	57223201	700	2	0.00081186	2	0.2	NA
DMR2:60537101	2	60537101	1600	2	0.00035886	5	0.3	OSBPL10
DMR2:67804701	2	67804701	500	2	0.00053012	2	0.4	
DMR2:74604401	2	74604401	600	2	0.00044348	5	0.8	
DMR2:76623901	2	76623901	400	2	0.00027441	1	0.2	
DMR2:81234601	2	81234601	600	2	0.00047515	3	0.5	
DMR2:91707301	2	91707301	1200	2	0.0006222	5	0.4	
DMR2:94031401	2	94031401	1600	3	0.00027513	4	0.2	NA
DMR2:96346101	2	96346101	900	2	0.00070456	5	0.5	
DMR2:108428901	2	108428901	300	2	0.00048682	0	0	
DMR2:118325701	2	118325701	300	2	0.00025038	1	0.3	TOX
DMR2:123640001	2	123640001	200	2	0.00029415	2	1	
DMR2:134150201	2	134150201	1500	2	0.00041082	0	0	NIPAL2

DMR2:144740601	2	144740601	400	2	0.00072872	1	0.2	FBXO32
DMR3:12996601	3	12996601	300	2	0.00014089	1	0.3	
DMR3:14233101	3	14233101	600	2	0.00016186	5	0.8	UBR2
DMR3:17642301	3	17642301	1800	2	0.00026862	6	0.3	
DMR3:20417201	3	20417201	1600	2	0.00029158	5	0.3	LCLAT1
DMR3:27575701	3	27575701	300	2	0.00051085	0	0	
DMR3:37439001	3	37439001	1500	2	0.00078426	1	0.06	PARK2
DMR3:41905401	3	41905401	1300	2	0.00041533	82	6.3	DLL1
DMR3:43634901	3	43634901	400	2	0.00067468	1	0.2	SIPA1L2
DMR3:47822101	3	47822101	1900	2	0.00077027	7	0.3	
DMR3:52144601	3	52144601	1100	2	0.00065509	5	0.4	
DMR3:53617101	3	53617101	900	2	0.00041859	2	0.2	SNX9
DMR3:57319601	3	57319601	2700	2	0.00018767	13	0.4	
DMR3:58282801	3	58282801	2600	2	0.00050741	10	0.3	
DMR3:62782501	3	62782501	1900	2	0.00033142	4	0.2	
DMR3:67318501	3	67318501	1800	2	0.00062474	8	0.4	
DMR3:72011901	3	72011901	400	2	0.00041523	1	0.2	
DMR3:78687001	3	78687001	4500	2	0.00024516	18	0.4	AKIRIN2
DMR3:85093801	3	85093801	300	2	0.00047423	1	0.3	FAM135A
DMR3:89692801	3	89692801	1000	2	0.00029415	0	0	BMP5
DMR3:92570201	3	92570201	1500	2	6.93E-05	7	0.4	CSMD1
DMR3:95496201	3	95496201	1700	2	0.00050741	4	0.2	PXDN
DMR3:102676701	3	102676701	400	2	0.00022277	2	0.5	
DMR4:30010001	4	30010001	500	2	0.00030323	3	0.6	RAPGEF2
DMR4:40007701	4	40007701	1100	2	0.00066563	3	0.2	
DMR4:46638801	4	46638801	2000	2	0.00038729	7	0.3	
DMR4:54765801	4	54765801	3000	2	0.00034629	7	0.2	
DMR4:57167101	4	57167101	1400	2	0.00043234	9	0.6	
DMR4:65124401	4	65124401	400	2	0.00033129	3	0.7	
DMR4A:4645401	4A	4645401	500	2	0.00020126	2	0.4	HDX
DMR5:11178401	5	11178401	2000	2	5.92E-05	60	3	
DMR5:17116201	5	17116201	500	2	0.00022882	0	0	NA
DMR5:19330701	5	19330701	600	3	0.0003843	0	0	
DMR5:20105501	5	20105501	300	2	5.25E-05	1	0.3	EXT2
DMR5:22209801	5	22209801	1100	2	0.00031993	6	0.5	AMBRA1
DMR5:25054401	5	25054401	400	2	0.00055585	1	0.2	UBR1
DMR5:25225401	5	25225401	300	2	0.00016997	0	0	
DMR5:29800501	5	29800501	500	2	0.00038454	1	0.2	RYR3
DMR5:36949201	5	36949201	2400	2	0.00052011	7	0.2	
DMR5:41558801	5	41558801	300	3	0.00047342	0	0	
DMR5:53829801	5	53829801	2100	2	0.00035467	9	0.4	
DMR6:5313901	6	5313901	900	2	0.00016145	2	0.2	C10orf107
DMR6:28395601	6	28395601	1100	2	0.0005434	5	0.4	NA
DMR6:29758301	6	29758301	300	2	0.00050694	0	0	
DMR6:32221801	6	32221801	1000	2	0.00020736	2	0.2	
DMR7:2821801	7	2821801	1300	2	0.00048644	9	0.6	IQCA1
DMR7:7413001	7	7413001	2500	2	0.0005313	11	0.4	
DMR7:8134501	7	8134501	1200	2	0.00051493	3	0.2	
DMR7:9858501	7	9858501	1400	2	0.00061522	8	0.5	
DMR7:27843701	7	27843701	2400	2	0.0004597	12	0.5	ZNF804A
DMR7:32399701	7	32399701	300	2	0.00029298	1	0.3	NA
DMR8:4830301	8	4830301	600	2	0.00043102	2	0.3	
DMR8:6695101	8	6695101	1500	2	9.23E-05	8	0.5	

DMR8:11398901	8	11398901	500	2	0.00074667	0	0	
DMR8:14216901	8	14216901	1200	2	0.00010694	0	0	
DMR8:14857401	8	14857401	200	2	0.00041888	0	0	
DMR8:25817401	8	25817401	3700	2	0.00045392	8	0.2	NA
DMR9:7480901	9	7480901	800	2	0.00038451	3	0.3	
DMR9:11711001	9	11711001	600	3	0.0003283	2	0.3	
DMR9:15033901	9	15033901	400	2	0.00017153	2	0.5	MB21D2
DMR10:7071601	10	7071601	300	2	0.00055586	1	0.3	
DMR10:8862401	10	8862401	2100	2	1.62E-05	14	0.6	TMOD2
DMR10:11607101	10	11607101	600	2	0.00066354	0	0	
DMR10:16087901	10	16087901	1400	2	0.00018825	9	0.6	
DMR11:14774301	11	14774301	1300	3	0.00051978	9	0.6	
DMR11:16902201	11	16902201	1800	2	0.00025038	3	0.1	
DMR11:17270401	11	17270401	2500	2	0.00014437	10	0.4	CDH8
DMR11:17582801	11	17582801	2200	2	0.00020408	5	0.2	
DMR11:18477501	11	18477501	2000	2	0.00032749	5	0.2	
DMR13:7280601	13	7280601	1500	2	0.00032367	3	0.2	
DMR13:10352201	13	10352201	500	2	0.00061683	2	0.4	
DMR13:13887701	13	13887701	3700	2	0.00052392	11	0.2	
DMR14:12007301	14	12007301	900	2	0.0006074	2	0.2	
DMR14:15125101	14	15125101	1300	2	0.00016353	11	0.8	NA
DMR15:8420501	15	8420501	900	2	0.0003794	1	0.1	TOP3B
DMR19:8815901	19	8815901	1100	2	0.0002476	3	0.2	GDPD1
DMR20:9459301	20	9459301	300	2	0.00037744	1	0.3	
DMR20:12224401	20	12224401	3000	2	0.0002881	67	2.2	
DMR26:1999201	26	1999201	1400	2	0.00022374	3	0.2	KLHL12
DMRZ:36983401	Z	36983401	1000	2	0.00068726	7	0.7	
DMRZ:42084601	Z	42084601	600	2	0.00049473	4	0.6	
DMRZ:45791301	Z	45791301	2200	2	0.00055305	8	0.3	
DMRZ:58234701	Z	58234701	5200	2	0.00016185	25	0.4	DENND4C
DMRZ:66392601	Z	66392601	600	2	0.00049011	0	0	
DMRUn:15781801	Un	15781801	700	2	0.00016516	3	0.4	
DMRUn:31802501	Un	31802501	600	2	0.00026326	5	0.8	
DMRUn:55354101	Un	55354101	200	2	0.00018537	1	0.5	
DMRUn:99227501	Un	99227501	400	2	0.00020246	3	0.7	
DMRUn:152715201	Un	152715201	200	2	0.00067783	1	0.5	NA

Supplemental Table S3B *G. fuliginosa* **Multiple-Window DMR Erythrocyte List**

DMR Name	Chr	Start	Length (bp)	# Sites	min P Value	CpG #	CpG Density (#/100bp)	Gene Association
DMR1:4372401	1	4372401	200	2	7.68E-06	7	3.5	
DMR1:4717301	1	4717301	2700	2	4.51E-05	17	0.6	
DMR1:8796601	1	8796601	200	2	0.0001461	0	0	
DMR1:15514901	1	15514901	700	2	7.48E-05	0	0	NA
DMR1:30194301	1	30194301	500	2	0.00020125	2	0.4	RNF149
DMR1:34868501	1	34868501	1400	2	0.00097878	7	0.5	
DMR1:49804201	1	49804201	400	2	1.76E-05	1	0.2	NA
DMR1:55662401	1	55662401	600	2	3.07E-05	3	0.5	
DMR1:55714201	1	55714201	1400	2	8.18E-05	7	0.5	
DMR1:56745701	1	56745701	600	2	1.26E-05	3	0.5	
DMR1:60281601	1	60281601	200	2	0.00024493	0	0	
DMR1:69585701	1	69585701	300	2	7.68E-06	2	0.6	LMO7
DMR1:79976701	1	79976701	300	2	0.00045066	0	0	NA
DMR1:80849001	1	80849001	200	2	0.00046822	0	0	FAT3
DMR1:80975101	1	80975101	300	2	7.87E-05	1	0.3	
DMR1:88272501	1	88272501	1400	2	0.00030252	15	1	
DMR1:88280001	1	88280001	400	2	0.00012253	0	0	
DMR1:91117201	1	91117201	300	2	0.00038876	2	0.6	
DMR1:91608001	1	91608001	1600	2	0.00013341	14	0.8	
DMR1:94220601	1	94220601	900	3	0.00012598	8	0.8	
DMR1:94822701	1	94822701	200	2	3.07E-05	3	1.5	LSAMP
DMR1:95189901	1	95189901	3700	2	0.00048961	24	0.6	
DMR1:109208501	1	109208501	2200	2	4.95E-05	9	0.4	
DMR1:116004201	1	116004201	200	2	0.00024493	0	0	
DMR1A:536501	1A	536501	2300	2	0.00026491	83	3.6	NET1
DMR1A:3291201	1A	3291201	800	2	8.20E-05	3	0.3	SFMBT2
DMR1A:19893901	1A	19893901	200	2	6.13E-05	1	0.5	
DMR1A:25828501	1A	25828501	300	2	0.00053859	2	0.6	PPP1R3A
DMR1A:37988101	1A	37988101	200	2	0.00036327	1	0.5	
DMR1A:38048601	1A	38048601	2000	2	3.55E-05	8	0.4	NAV3
DMR1A:48081501	1A	48081501	800	2	0.00044329	2	0.2	
DMR1A:50021401	1A	50021401	700	2	0.00075736	6	0.8	CACNA1I
DMR1A:59233601	1A	59233601	1600	2	7.32E-05	2	0.1	
DMR1A:60465701	1A	60465701	300	2	3.07E-05	1	0.3	SOX5
DMR1A:67777301	1A	67777301	300	2	0.00012253	0	0	PLEKHA5
DMR1A:70315301	1A	70315301	1100	2	7.89E-05	11	1	
DMR1A:72702301	1A	72702301	300	2	0.00012253	1	0.3	
DMR1A:73391301	1A	73391301	1700	2	0.00068727	9	0.5	
DMR2:5091301	2	5091301	3300	2	6.37E-05	16	0.4	
DMR2:6286001	2	6286001	300	2	0.00029475	4	1.3	
DMR2:9116901	2	9116901	1200	2	0.00055161	4	0.3	
DMR2:11574001	2	11574001	1200	2	0.00012903	2	0.1	
DMR2:13073201	2	13073201	1100	2	8.15E-05	1	0.09	
DMR2:13156501	2	13156501	400	2	0.00023991	1	0.2	
DMR2:14049001	2	14049001	1100	2	0.00075338	1	0	
DMR2:14069501	2	14069501	200	2	0.00010969	0	0	
DMR2:16620301	2	16620301	400	2	0.00038799	2	0.5	
DMR2:18882501	2	18882501	1200	2	0.00048961	15	1.2	

DMR2:21606201	2	21606201	300	2	4.12E-05	0	0	NA
DMR2:30149401	2	30149401	200	2	0.00051448	1	0.5	
DMR2:34396601	2	34396601	200	2	1.68E-07	0	0	
DMR2:39353301	2	39353301	700	2	0.00040467	5	0.7	
DMR2:49454001	2	49454001	300	2	3.72E-05	0	0	
DMR2:66551001	2	66551001	400	2	0.00022321	2	0.5	EEPD1
DMR2:69476301	2	69476301	400	2	0.00032254	1	0.2	
DMR2:71040901	2	71040901	300	2	2.32E-05	2	0.6	
DMR2:71432601	2	71432601	500	2	0.00083302	1	0.2	
DMR2:75127401	2	75127401	400	2	0.00039202	0	0	
DMR2:82366501	2	82366501	800	2	7.33E-05	4	0.5	
DMR2:84797701	2	84797701	900	2	0.00017431	8	0.8	MYH6
DMR2:87015801	2	87015801	200	2	0.00021576	0	0	
DMR2:88439501	2	88439501	600	2	0.00048961	2	0.3	FHOD3
DMR2:88582201	2	88582201	200	2	9.21E-05	0	0	C18orf21
DMR2:93255301	2	93255301	800	2	0.00029452	3	0.3	
DMR2:94902101	2	94902101	1200	2	0.0006423	12	1	
DMR2:97747101	2	97747101	200	2	8.18E-05	0	0	
DMR2:100091801	2	100091801	200	2	0.00020101	0	0	
DMR2:102792501	2	102792501	200	2	0.00024493	0	0	GNAL
DMR2:107228101	2	107228101	1500	2	0.00012253	6	0.4	
DMR2:109112201	2	109112201	300	2	8.82E-05	3	1	OSBPL1A
DMR2:110670701	2	110670701	500	2	0.00026973	2	0.4	CDH2
DMR2:112067801	2	112067801	1100	2	4.25E-05	4	0.3	
DMR2:115575001	2	115575001	300	2	0.00023639	0	0	
DMR2:118036701	2	118036701	300	2	7.67E-05	2	0.6	
DMR2:119271201	2	119271201	1800	2	0.00026098	8	0.4	
DMR2:120341401	2	120341401	200	2	0.00052692	4	2	
DMR2:121488201	2	121488201	1900	2	8.26E-05	9	0.4	NA
DMR2:132400801	2	132400801	200	2	0.00036063	0	0	CDH17
DMR2:133334401	2	133334401	1300	2	0.00018133	74	5.6	
DMR2:133357301	2	133357301	1700	2	0.00048961	7	0.4	
DMR2:138528101	2	138528101	200	2	0.00075338	1	0.5	NA
DMR2:140587701	2	140587701	200	2	0.00027586	0	0	
DMR2:141908201	2	141908201	400	2	0.00014206	0	0	EIF3H
DMR2:144011701	2	144011701	200	2	0.00031234	4	2	
DMR2:144085301	2	144085301	300	2	0.00046914	1	0.3	
DMR2:144294901	2	144294901	200	2	3.84E-06	0	0	
DMR2:149096501	2	149096501	300	2	0.00015574	0	0	
DMR2:151126601	2	151126601	1100	2	1.60E-05	15	1.3	FAM135B
DMR2:151971901	2	151971901	1200	2	0.0002323	5	0.4	KCNK9
DMR2:153992101	2	153992101	700	2	0.00012885	7	1	
DMR2:155785401	2	155785401	2000	2	0.00058204	28	1.4	ZC3H3
DMR3:3457301	3	3457301	500	2	3.09E-05	1	0.2	
DMR3:5223801	3	5223801	400	2	0.00066758	1	0.2	TASP1
DMR3:5355701	3	5355701	200	2	0.00027129	0	0	
DMR3:7621301	3	7621301	1700	2	0.00019112	38	2.2	
DMR3:9086001	3	9086001	400	2	0.00027586	0	0	
DMR3:13404601	3	13404601	200	2	0.00040864	0	0	
DMR3:21546801	3	21546801	5100	2	0.00016536	21	0.4	FOXN3
DMR3:26913201	3	26913201	1600	2	4.21E-05	13	0.8	

DMR3:32739301	3	32739301	400	3	3.14E-07	2	0.5	
DMR3:47496101	3	47496101	500	2	0.00027586	3	0.6	UST
DMR3:54732001	3	54732001	200	2	0.00097878	0	0	NOX3
DMR3:58937401	3	58937401	500	2	3.37E-05	2	0.4	
DMR3:64095701	3	64095701	600	2	2.29E-05	1	0.1	
DMR3:68932001	3	68932001	1600	2	0.00016801	3	0.1	
DMR3:70631201	3	70631201	200	2	0.00014652	0	0	PRDM1
DMR3:72222201	3	72222201	300	2	0.00086697	2	0.6	
DMR3:74530101	3	74530101	500	2	3.50E-06	3	0.6	
DMR3:77533001	3	77533001	3000	2	0.00028017	22	0.7	
DMR3:89074901	3	89074901	1000	2	3.07E-05	2	0.2	PRIM2
DMR3:89714201	3	89714201	800	2	2.64E-05	4	0.5	BMP5
DMR3:90528101	3	90528101	800	2	0.00048961	3	0.3	GCLC
DMR3:95828401	3	95828401	400	2	0.00029828	5	1.2	
DMR3:102264401	3	102264401	700	2	0.00027586	0	0	
DMR3:103500401	3	103500401	1400	3	6.87E-07	7	0.5	
DMR3:105780201	3	105780201	200	2	0.00024493	0	0	
DMR3:110489001	3	110489001	1300	2	4.53E-05	5	0.3	NA
DMR4:5567801	4	5567801	200	2	0.0003297	0	0	SPATA5
DMR4:6440901	4	6440901	2000	2	5.60E-05	16	0.8	FAT4
DMR4:11522601	4	11522601	300	2	0.00028556	1	0.3	ARHGAP10
DMR4:21854601	4	21854601	400	2	0.00024493	3	0.7	
DMR4:23287201	4	23287201	1200	2	0.00044914	10	0.8	LEF1
DMR4:30011901	4	30011901	1000	2	7.46E-05	4	0.4	RAPGEF2
DMR4:35035201	4	35035201	300	2	0.00018333	0	0	
DMR4:38514801	4	38514801	1700	2	5.70E-05	10	0.5	ENPP6
DMR4:47123001	4	47123001	300	2	0.00015385	0	0	NA
DMR4:54085101	4	54085101	400	2	0.00058308	0	0	
DMR4:59851201	4	59851201	500	2	0.00097878	2	0.4	
DMR4A:3765401	4A	3765401	1100	2	0.00053963	5	0.4	
DMR4A:6082701	4A	6082701	1000	2	0.00071146	17	1.7	
DMR4A:9160301	4A	9160301	1000	2	2.37E-05	1	0.1	
DMR4A:9445601	4A	9445601	500	2	0.00012253	2	0.4	MCTS1;AKAP14
DMR4A:12525201	4A	12525201	1100	2	0.00012253	1	0.09	
DMR4A:12646701	4A	12646701	200	2	0.00087502	1	0.5	
DMR4A:13403801	4A	13403801	400	2	0.00048961	1	0.2	
DMR4A:13875801	4A	13875801	500	2	0.00026475	3	0.6	
DMR4A:17382501	4A	17382501	400	2	1.81E-05	3	0.7	
DMR4A:17926601	4A	17926601	900	2	1.81E-05	6	0.6	
DMR4A:18342901	4A	18342901	200	2	0.00060352	0	0	
DMR5:143201	5	143201	2400	2	0.00024493	14	0.5	NELL1
DMR5:5322501	5	5322501	300	2	0.00011009	4	1.3	
DMR5:7862001	5	7862001	1000	2	9.51E-05	23	2.3	NA
DMR5:15807101	5	15807101	1100	2	0.00059893	2	0.1	RPLP2
DMR5:23278601	5	23278601	2800	2	0.00091069	36	1.2	
DMR5:23337901	5	23337901	400	2	0.00077175	1	0.2	INO80
DMR5:26249001	5	26249001	200	2	4.81E-05	1	0.5	
DMR5:28428001	5	28428001	2400	2	4.03E-05	5	0.2	
DMR5:28566801	5	28566801	200	2	0.00024493	0	0	GPHN
DMR5:28908101	5	28908101	600	2	0.00044855	3	0.5	
DMR5:31294901	5	31294901	2400	3	0.00023909	10	0.4	

DMR5:34525601	5	34525601	1000	2	0.00048961	4	0.4	
DMR5:36924001	5	36924001	300	2	0.00098308	3	1	
DMR5:40106301	5	40106301	1500	2	0.00013108	15	1	NA
DMR5:44895801	5	44895801	800	2	0.00097878	3	0.3	TTC7B
DMR5:48919901	5	48919901	800	2	0.00029446	6	0.7	NA
DMR5:53365301	5	53365301	300	2	0.00014568	0	0	
DMR5:53490201	5	53490201	1600	2	0.00046167	5	0.3	CEP170B
DMR5:53560501	5	53560501	200	2	0.00071995	4	2	
DMR5:54589001	5	54589001	400	2	2.63E-05	5	1.2	BRF1
DMR5:54610101	5	54610101	1000	2	0.00034145	17	1.7	BRF1
DMR5:57026201	5	57026201	400	2	9.21E-05	1	0.2	
DMR5:58011201	5	58011201	300	2	5.69E-05	5	1.6	
DMR5:58386501	5	58386501	1000	3	1.54E-05	16	1.6	TMEM260
DMR6:16318101	6	16318101	2600	2	1.13E-06	15	0.5	
DMR6:25933601	6	25933601	1600	4	6.13E-05	8	0.5	NA
DMR6:36172101	6	36172101	1200	2	6.29E-05	6	0.5	
DMR7:8264601	7	8264601	200	2	4.35E-05	1	0.5	
DMR7:10812401	7	10812401	3400	2	5.96E-05	21	0.6	SLC4A3
DMR7:11920001	7	11920001	200	2	4.37E-05	1	0.5	IFIH1
DMR7:17993901	7	17993901	1400	2	7.96E-05	8	0.5	
DMR7:26104901	7	26104901	300	2	0.00051055	7	2.3	ORMDL1
DMR7:36578601	7	36578601	1000	2	0.00029064	17	1.7	
DMR7:39775501	7	39775501	200	2	4.89E-07	2	1	
DMR8:1072401	8	1072401	1700	2	7.68E-06	7	0.4	
DMR8:4542101	8	4542101	300	2	5.51E-06	1	0.3	CRB1
DMR8:10439801	8	10439801	1300	3	0.00012253	15	1.1	RPL5;SNORD21
DMR8:11860301	8	11860301	200	2	0.00018568	1	0.5	
DMR8:12439401	8	12439401	1100	2	6.13E-05	4	0.3	
DMR8:13603601	8	13603601	200	2	8.65E-05	1	0.5	
DMR8:15006401	8	15006401	300	2	0.00052699	0	0	
DMR8:19866501	8	19866501	1100	2	0.00017471	13	1.1	
DMR8:22901801	8	22901801	300	2	6.51E-05	2	0.6	
DMR8:23883401	8	23883401	2400	3	0.00013798	22	0.9	
DMR8:24123401	8	24123401	600	2	0.00018994	1	0.1	
DMR8:24651101	8	24651101	200	2	0.00052459	0	0	
DMR8:24810801	8	24810801	800	2	0.00048961	0	0	
DMR8:24850501	8	24850501	300	2	9.01E-05	1	0.3	
DMR8:26306401	8	26306401	1100	2	0.00010926	5	0.4	AK4
DMR9:659301	9	659301	300	2	0.00047856	1	0.3	
DMR9:5238901	9	5238901	1000	2	0.00052077	1	0.1	NA
DMR9:7457601	9	7457601	300	2	0.00014515	1	0.3	SPSB4
DMR9:8221501	9	8221501	300	2	0.00040869	1	0.3	
DMR9:14240601	9	14240601	300	2	9.65E-05	4	1.3	
DMR9:14369701	9	14369701	400	2	0.00021154	1	0.2	
DMR9:19526001	9	19526001	600	2	0.00012253	1	0.1	
DMR9:20093101	9	20093101	1800	2	1.03E-05	8	0.4	MFN1
DMR9:20439401	9	20439401	1000	2	0.00048961	3	0.3	
DMR9:22520301	9	22520301	200	2	5.23E-05	1	0.5	
DMR9:24201901	9	24201901	900	2	0.00059108	1	0.1	
DMR9:25227801	9	25227801	2600	2	0.000835	15	0.5	
DMR9:25731701	9	25731701	300	2	0.00097878	1	0.3	MFSD1

DMR9:26210101	9	26210101	300	2	3.16E-05	2	0.6	NA
DMR9:27015201	9	27015201	200	2	0.00038638	1	0.5	PFN2
DMR10:7027301	10	7027301	1900	2	1.36E-05	7	0.3	CGNL1
DMR10:7163301	10	7163301	300	2	5.03E-05	1	0.3	
DMR10:10285701	10	10285701	200	2	5.76E-05	2	1	
DMR10:13256501	10	13256501	500	2	0.00010529	2	0.4	DET1
DMR10:13316301	10	13316301	1600	2	0.00014568	11	0.6	
DMR10:15606601	10	15606601	200	2	5.72E-05	3	1.5	
DMR10:17519401	10	17519401	3400	2	4.07E-05	10	0.2	
DMR10:17985101	10	17985101	300	2	0.00023797	5	1.6	ALDH1A3
DMR10:18730201	10	18730201	300	2	0.00086225	1	0.3	DENND4A
DMR11:3177001	11	3177001	300	2	5.05E-06	1	0.3	
DMR11:3675801	11	3675801	1100	3	0.00042951	1	0.09	
DMR11:8358501	11	8358501	1200	2	7.67E-05	1	0.08	
DMR11:9094101	11	9094101	500	2	0.00070224	1	0.2	
DMR11:9926601	11	9926601	300	2	3.07E-05	1	0.3	
DMR11:10915301	11	10915301	1600	2	0.00057165	9	0.5	BANP
DMR11:11887801	11	11887801	700	2	0.00035386	3	0.4	
DMR11:15791101	11	15791101	1200	2	0.00048961	3	0.2	
DMR11:15948901	11	15948901	200	2	0.00040956	2	1	
DMR11:17071401	11	17071401	1700	2	0.00024067	12	0.7	
DMR11:19202501	11	19202501	1600	2	7.67E-05	5	0.3	NA
DMR12:424701	12	424701	1300	2	0.00060191	7	0.5	NA
DMR12:1074001	12	1074001	300	2	0.00011721	0	0	
DMR12:2681801	12	2681801	200	2	0.00024493	0	0	
DMR12:15878101	12	15878101	300	2	1.30E-05	3	1	
DMR12:16143901	12	16143901	2300	2	1.65E-05	17	0.7	FAM19A1
DMR12:18973401	12	18973401	1200	2	6.03E-06	12	1	
DMR12:19028901	12	19028901	400	2	0.00010033	1	0.2	
DMR12:19307701	12	19307701	300	2	0.00014568	6	2	
DMR12:20529201	12	20529201	200	2	3.84E-06	4	2	
DMR12:21383601	12	21383601	1700	2	0.00023678	6	0.3	
DMR13:4235701	13	4235701	200	2	4.92E-05	1	0.5	EBF1
DMR13:4487901	13	4487901	200	2	0.00022422	3	1.5	
DMR13:5482801	13	5482801	1100	2	5.36E-05	5	0.4	
DMR13:6425801	13	6425801	600	2	0.0003907	1	0.1	
DMR13:8068401	13	8068401	1700	2	0.00014568	12	0.7	
DMR13:9793401	13	9793401	600	2	0.00059893	3	0.5	
DMR13:11390101	13	11390101	200	2	0.00070224	1	0.5	
DMR13:16263501	13	16263501	700	2	0.00044215	15	2.1	
DMR13:16826401	13	16826401	2700	2	1.85E-05	378	14	
DMR14:922901	14	922901	1800	2	0.00010341	11	0.6	
DMR14:1865701	14	1865701	500	2	0.00097974	3	0.6	RAB11FIP3
DMR14:7746201	14	7746201	200	2	0.00024493	1	0.5	MYH11
DMR14:12143301	14	12143301	1400	2	0.00043873	13	0.9	
DMR15:3320201	15	3320201	200	2	0.00048961	3	1.5	
DMR15:9116301	15	9116301	300	2	9.07E-05	1	0.3	
DMR15:10710801	15	10710801	1400	2	0.00044301	4	0.2	
DMR15:11293101	15	11293101	2300	2	0.00010906	20	0.8	EIF4ENIF1
DMR17:6864401	17	6864401	1100	2	0.00023253	13	1.1	PRRC2B
DMR17:11018301	17	11018301	200	2	0.00066212	34	17	PBX3

DMR18:126301	18	126301	1400	2	8.78E-05	6	0.4	
DMR18:1033201	18	1033201	700	2	0.00050364	4	0.5	NPLOC4
DMR18:3560201	18	3560201	1300	2	6.13E-05	6	0.4	
DMR18:8856901	18	8856901	800	2	0.00025713	12	1.5	MRPS7
DMR18:9121601	18	9121601	200	2	6.13E-05	2	1	
DMR19:5744201	19	5744201	600	2	1.26E-05	2	0.3	
DMR19:7251301	19	7251301	1000	2	6.13E-05	5	0.5	
DMR19:8830401	19	8830401	1700	2	0.00013425	13	0.7	
DMR19:10177801	19	10177801	200	2	7.70E-05	2	1	ACACA
DMR20:12291301	20	12291301	200	2	0.00063673	1	0.5	NA
DMR20:12433001	20	12433001	1600	2	2.27E-05	20	1.2	VAPB
DMR20:13029701	20	13029701	1000	2	0.00097878	9	0.9	BMP7
DMR21:149301	21	149301	300	2	5.95E-05	3	1	
DMR21:1575701	21	1575701	200	2	1.18E-05	1	0.5	
DMR21:3489101	21	3489101	200	2	6.13E-05	0	0	
DMR21:3618001	21	3618001	2100	2	0.00024527	26	1.2	SKI
DMR21:3922101	21	3922101	3700	2	0.00027586	32	0.8	NA
DMR21:4701101	21	4701101	400	2	1.53E-05	3	0.7	
DMR21:4941801	21	4941801	200	2	0.00011491	0	0	UBE4B
DMR21:5494101	21	5494101	1400	2	0.00031974	7	0.5	RERE
DMR22:3011601	22	3011601	200	2	0.0003452	3	1.5	NA
DMR23:2525401	23	2525401	400	2	0.00060005	4	1	FAM76A
DMR24:1935801	24	1935801	200	2	0.00059565	5	2.5	NA
DMR26:3370501	26	3370501	2300	2	0.0002477	22	0.9	RAP1A
DMR26:4774101	26	4774101	600	2	0.00015107	4	0.6	NA
DMR27:3371401	27	3371401	300	2	0.00052077	1	0.3	TLK2
DMR27:3902601	27	3902601	1100	2	0.00012399	7	0.6	
DMR28:538701	28	538701	600	2	0.00031694	6	1	NA
DMRZ:7053001	Z	7053001	200	2	0.00023076	2	1	
DMRZ:8349501	Z	8349501	300	2	0.00023147	0	0	NA
DMRZ:11290201	Z	11290201	400	2	0.00032687	1	0.2	
DMRZ:22572801	Z	22572801	400	2	0.00052077	1	0.2	
DMRZ:33485401	Z	33485401	200	2	0.00014568	1	0.5	SETBP1
DMRZ:36664001	Z	36664001	900	2	0.00048961	2	0.2	
DMRZ:38648801	Z	38648801	300	2	0.00014568	1	0.3	
DMRZ:38745301	Z	38745301	1700	2	0.00010258	12	0.7	
DMRZ:38864501	Z	38864501	400	2	0.00046444	2	0.5	
DMRZ:39510601	Z	39510601	200	2	2.24E-05	1	0.5	
DMRZ:39662301	Z	39662301	3300	2	0.00034602	32	0.9	NA
DMRZ:40335601	Z	40335601	200	2	0.00065736	1	0.5	
DMRZ:40862001	Z	40862001	400	2	0.00052901	2	0.5	
DMRZ:46606301	Z	46606301	700	3	0.00011601	22	3.1	FST
DMRZ:49883001	Z	49883001	300	2	2.14E-06	3	1	IPO11
DMRZ:53351401	Z	53351401	1700	2	1.88E-05	6	0.3	
DMRZ:54426901	Z	54426901	1100	2	0.00048728	3	0.2	
DMRZ:66310201	Z	66310201	500	2	6.13E-05	2	0.4	FCHO2
DMRUn:1724101	Un	1724101	200	2	5.20E-06	6	3	
DMRUn:7287501	Un	7287501	1300	2	0.00010725	12	0.9	
DMRUn:9275501	Un	9275501	200	2	1.92E-08	0	0	
DMRUn:13837801	Un	13837801	900	2	0.00010152	5	0.5	
DMRUn:19838801	Un	19838801	1700	2	1.58E-06	8	0.4	

DMRUn:26079901	Un	26079901	1000	2	0.00014707	7	0.7
DMRUn:28474101	Un	28474101	200	2	0.00052077	2	1
DMRUn:44797501	Un	44797501	300	2	0.00024493	3	1
DMRUn:63885501	Un	63885501	200	2	0.00028473	1	0.5
DMRUn:81577201	Un	81577201	500	2	0.00029103	1	0.2
DMRUn:101350901	Un	101350901	500	2	8.15E-07	6	1.2
DMRUn:102335201	Un	102335201	200	2	6.13E-05	0	0
DMRUn:111843701	Un	111843701	200	2	0.00024493	0	0
DMRUn:125483701	Un	125483701	200	2	0.00034005	0	0
DMRUn:128057501	Un	128057501	500	2	6.11E-05	15	3
DMRUn:130868301	Un	130868301	200	2	0.00012253	3	1.5
DMRUn:133170701	Un	133170701	300	2	3.07E-05	2	0.6
DMRUn:138655101	Un	138655101	200	2	0.00027129	2	1
DMRUn:139521501	Un	139521501	1800	2	1.03E-05	20	1.1
DMRUn:141968301	Un	141968301	200	2	0.00026973	1	0.5
DMRUn:147075801	Un	147075801	2400	2	0.00024345	20	0.8
DMRUn:148458601	Un	148458601	400	2	7.68E-06	1	0.2
DMRUn:151192001	Un	151192001	200	2	0.00013794	0	0
DMRUn:158423601	Un	158423601	300	2	0.00034419	2	0.6
DMRUn:163874501	Un	163874501	200	2	0.00040692	1	0.5
DMRUn:165781501	Un	165781501	1300	2	3.66E-05	10	0.7
