# Supplementary material for: Epigenetic variation between urban and rural populations of Darwin’s finches
Source: BMC Evol Biol. 2017 Aug 24;17:183. doi: 10.1186/s12862-017-1025-9 (PMC5569522; doi:10.1186/s12862-017-1025-9)
Supplement: Supplementary file 6 — Description of DMR clusters detected in G. fortis sperm (A) and erythrocytes (B) and G. fuliginosa sperm (C) and erythrocytes (D). Description includes DMR in cluster, chromosome number, cluster start site, cluster stop site, length in bp, and minimum p-value. (PDF 103 kb) [file 12862_2017_1025_MOESM6_ESM.pdf]

# Supplemental Table S4
# Cluster Analysis Lists and Number

**Supplemental Table S4A *G. fortis***
**DMR Sperm List**

| DMR in Cluster | Chr | Start | Stop | Length (bp) | Min p-value |
|---|---|---|---|---|---|
| DMR10:14143801;DMR10:14466301;DMR10:15617101;DMR10:17182001 | 10 | 12500000 | 17550000 | 5050000 | 2.26E-06 |
| DMR11:14550401;DMR11:16380101 | 11 | 14400000 | 16500000 | 2100000 | 0.030 |
| DMR12:4785401;DMR12:5901001 | 12 | 3950000 | 6700000 | 2750000 | 0.030 |
| DMR14:1181901;DMR14:3090701 | 14 | 1100000 | 3100000 | 2.00E+06 | 0.030 |
| DMR15:2627801;DMR15:2915401 | 15 | 950000 | 4550000 | 3600000 | 0.030 |
| DMR18:991001;DMR18:1081001;DMR18:1084101 | 18 | 50000 | 3.00E+06 | 2950000 | 2.26E-06 |
| DMR2:1016201;DMR2:1382701 | 2 | 50000 | 3.00E+06 | 2950000 | 0.030 |
| DMR2:57021501;DMR2:57355101 | 2 | 55400000 | 58950000 | 3550000 | 0.030 |
| DMR20:353601;DMR20:553601;DMR20:1139801 | 20 | 50000 | 2550000 | 2500000 | 0.030 |
| DMR20:12107601;DMR20:13641501 | 20 | 11650000 | 14050000 | 2400000 | 0.030 |
| DMR28:3706001;DMR28:4853401 | 28 | 2900000 | 5650000 | 2750000 | 0.030 |
| DMR3:110673201;DMR3:111805401;DMR3:112515501 | 3 | 109850000 | 113750000 | 3900000 | 2.26E-06 |
| DMR4:5948301;DMR4:6462901 | 4 | 4500000 | 7850000 | 3350000 | 0.030 |
| DMR4A:17926101;DMR4A:19342701 | 4A | 17350000 | 19850000 | 2500000 | 0.030 |
| DMR7:1620001;DMR7:2068201 | 7 | 1.00E+05 | 3550000 | 3450000 | 0.030 |
| DMRUn:37518901;DMRUn:38440401 | Un | 36450000 | 39500000 | 3050000 | 0.030 |
| DMRUn:64017401;DMRUn:65244001 | Un | 63250000 | 6.60E+07 | 2750000 | 0.030 |
| DMRUn:70786601;DMRUn:70799501 | Un | 68850000 | 72750000 | 3900000 | 0.030 |
| DMRUn:93117201;DMRUn:93590701 | Un | 91600000 | 95100000 | 3500000 | 0.030 |
| DMRUn:116431501;DMRUn:116729701 | Un | 114750000 | 118400000 | 3650000 | 0.030 |
| DMRUn:142781801;DMRUn:144276901 | Un | 142300000 | 144750000 | 2450000 | 0.030 |
| DMRUn:155480601;DMRUn:156914801 | Un | 154950000 | 157400000 | 2450000 | 0.030 |

**Supplemental Table S4B *G. fortis* DMR**
**Cluster Erythrocyte List**

| DMR in Cluster | Chr | Start | Stop | Length (bp) | Min p-value |
|---|---|---|---|---|---|
| DMR1:15978201;DMR1:17714201 | 1 | 15750000 | 17950000 | 2200000 | 0.020 |
| DMR1:93094801;DMR1:93731001 | 1 | 91750000 | 95050000 | 3300000 | 0.020 |
| DMR1:116098201;DMR1:117036401 | 1 | 115050000 | 1.18E+08 | 2950000 | 0.020 |
| DMR10:11658001;DMR10:11904101 | 10 | 9950000 | 13600000 | 3650000 | 0.020 |
| DMR12:9477701;DMR12:10036401;DMR12:11939801 | 12 | 8050000 | 11950000 | 3900000 | 0.020 |
| DMR14:8553401;DMR14:10140501 | 14 | 8150000 | 10500000 | 2350000 | 0.020 |
| DMR15:12868101;DMR15:13976501;DMR15:14137901 | 15 | 1.20E+07 | 15900000 | 3900000 | 4.77E-07 |
| DMR1A:37330801;DMR1A:37641201 | 1A | 35650000 | 39300000 | 3650000 | 0.020 |
| DMR1A:46028501;DMR1A:47095601 | 1A | 45100000 | 4.80E+07 | 2900000 | 0.020 |
| DMR1A:68987201;DMR1A:70257101 | 1A | 68300000 | 70900000 | 2600000 | 0.020 |
| DMR2:106386901;DMR2:106836801 | 2 | 104850000 | 108300000 | 3450000 | 0.020 |
| DMR20:4414401;DMR20:5555701;DMR20:6871001 | 20 | 3600000 | 7500000 | 3900000 | 0.020 |
| DMR26:3225201;DMR26:4154601 | 26 | 2200000 | 5150000 | 2950000 | 0.020 |
| DMR3:16726301;DMR3:17261701 | 3 | 15300000 | 18700000 | 3400000 | 0.020 |
| DMR3:24857301;DMR3:26735901 | 3 | 24750000 | 26850000 | 2100000 | 0.0202 |
| DMR3:78517101;DMR3:78536501 | 3 | 76550000 | 80450000 | 3900000 | 0.020 |
| DMR5:2675901;DMR5:2884401 | 5 | 9.00E+05 | 4600000 | 3700000 | 0.020 |
| DMR6:24244501;DMR6:24443901 | 6 | 22450000 | 26150000 | 3700000 | 0.020 |
| DMR7:7103301;DMR7:7578001 | 7 | 5600000 | 9100000 | 3500000 | 0.020 |
| DMR7:37235801;DMR7:37271201;DMR7:38670601 | 7 | 35300000 | 39200000 | 3900000 | 4.77E-07 |
| DMRUn:38098901;DMRUn:39304701 | Un | 37350000 | 40050000 | 2700000 | 0.020 |
| DMRUn:166943301;DMRUn:167343801 | Un | 165350000 | 168850000 | 3500000 | 0.020 |
| DMRZ:45594501;DMRZ:45691301 | Z | 43700000 | 47500000 | 3800000 | 0.020 |

**Supplemental Table S4C *G. fuliginosa***
**Cluster Sperm List**

| DMR in Cluster | Chr | Start | Stop | Length | Min p-value |
|---|---|---|---|---|---|
| DMR1:89065301;DMR1:89740001 | 1 | 87750000 | 9.10E+07 | 3250000 | 0.007 |
| DMR10:7071601;DMR10:8862401 | 10 | 6900000 | 9.00E+06 | 2100000 | 0.007 |
| DMR11:16902201;DMR11:17270401;DMR11:17582801;DMR11:18477501 | 11 | 15300000 | 19500000 | 4200000 | 7.22E-18 |
| DMR1A:5308001;DMR1A:6286301 | 1A | 4300000 | 7300000 | 3000000 | 0.007 |
| DMR1A:11577701;DMR1A:13180201 | 1A | 11200000 | 13550000 | 2350000 | 0.007 |
| DMR1A:22795501;DMR1A:24114101;DMR1A:25971701;DMR1A:27064701 | 1A | 22150000 | 27950000 | 5800000 | 0.007 |
| DMR1A:59545101;DMR1A:61248301 | 1A | 59250000 | 61450000 | 2200000 | 0.007 |
| DMR2:7094701;DMR2:8947601 | 2 | 6950000 | 9050000 | 2100000 | 0.007 |
| DMR2:19980701;DMR2:21027601;DMR2:21459501;DMR2:22412201 | 2 | 19050000 | 23450000 | 4400000 | 0.007 |
| DMR2:46614201;DMR2:47305801 | 2 | 45350000 | 48550000 | 3200000 | 0.007 |
| DMR3:12996601;DMR3:14233101 | 3 | 12250000 | 14950000 | 2700000 | 0.007 |
| DMR3:41905401;DMR3:43634901 | 3 | 41650000 | 43900000 | 2250000 | 0.007 |
| DMR3:52144601;DMR3:53617101 | 3 | 51650000 | 54100000 | 2450000 | 0.007 |
| DMR3:57319601;DMR3:58282801 | 3 | 56300000 | 59250000 | 2950000 | 0.007 |
| DMR5:19330701;DMR5:20105501 | 5 | 18150000 | 21300000 | 3150000 | 0.007 |
| DMR5:25054401;DMR5:25225401 | 5 | 23250000 | 2.70E+07 | 3750000 | 0.007 |
| DMR6:28395601;DMR6:29758301 | 6 | 27800000 | 30300000 | 2500000 | 0.007 |
| DMR7:7413001;DMR7:8134501;DMR7:9858501 | 7 | 6150000 | 10050000 | 3900000 | 0.007 |
| DMR8:4830301;DMR8:6695101 | 8 | 4700000 | 6800000 | 2100000 | 0.007 |
| DMR8:14216901;DMR8:14857401 | 8 | 12900000 | 16150000 | 3250000 | 0.007 |

**Supplemental Table S4D *G. fuliginosa***
**Cluster Erythrocyte List**

| DMR in Cluster | Chr | Start | Stop | Length (bp) | Min p-value |
|---|---|---|---|---|---|
| DMR1:55662401;DMR1:55714201;DMR1:56745701 | 1 | 54750000 | 57650000 | 2900000 | 0.017 |
| DMR1:79976701;DMR1:80849001;DMR1:80975101 | 1 | 7.90E+07 | 81950000 | 2950000 | 0.017 |
| DMR1:94220601;DMR1:94822701;DMR1:95189901 | 1 | 93200000 | 96150000 | 2950000 | 0.017 |
| DMR10:17519401;DMR10:17985101;DMR10:18730201 | 10 | 16750000 | 19450000 | 2700000 | 0.017 |
| DMR11:8358501;DMR11:9094101;DMR11:9926601;DMR11:10915301;DMR11:11887801 | 11 | 7950000 | 11900000 | 3950000 | 0.017 |
| DMR11:15791101;DMR11:15948901;DMR11:17071401 | 11 | 15100000 | 17700000 | 2600000 | 0.017 |
| DMR12:18973401;DMR12:19028901;DMR12:19307701;DMR12:20529201 | 12 | 17350000 | 20950000 | 3600000 | 0.000144629 |
| DMR13:4235701;DMR13:4487901;DMR13:5482801 | 13 | 3500000 | 6200000 | 2700000 | 0.017 |
| DMR2:11574001;DMR2:13073201;DMR2:13156501;DMR2:14049001;DMR2:14069501 | 2 | 11200000 | 15150000 | 3950000 | 0.017 |
| DMR2:69476301;DMR2:71040901;DMR2:71432601 | 2 | 69450000 | 71450000 | 2.00E+06 | 0.017 |
| DMR2:87015801;DMR2:88439501;DMR2:88582201 | 2 | 86600000 | 8.90E+07 | 2400000 | 0.017 |
| DMR2:132400801;DMR2:133334401;DMR2:133357301 | 2 | 131400000 | 134400000 | 3.00E+06 | 0.017 |
| DMR2:144011701;DMR2:144085301;DMR2:144294901 | 2 | 142300000 | 145950000 | 3650000 | 0.017 |
| DMR20:12291301;DMR20:12433001;DMR20:13029701 | 20 | 11050000 | 14200000 | 3150000 | 0.017 |
| DMR21:3489101;DMR21:3618001;DMR21:3922101;DMR21:4701101;DMR21:4941801;DMR21:5494101 | 21 | 1950000 | 6650000 | 4700000 | 1.67E-07 |
| DMR3:3457301;DMR3:5223801;DMR3:5355701 | 3 | 3400000 | 5450000 | 2050000 | 0.017 |
| DMR3:89074901;DMR3:89714201;DMR3:90528101 | 3 | 88550000 | 9.10E+07 | 2450000 | 0.017 |
| DMR4A:12525201;DMR4A:12646701;DMR4A:13403801;DMR4A:13875801 | 4A | 11450000 | 14600000 | 3150000 | 0.017 |
| DMR4A:17382501;DMR4A:17926601;DMR4A:18342901 | 4A | 16350000 | 19300000 | 2950000 | 0.017 |
| DMR5:28428001;DMR5:28566801;DMR5:28908101 | 5 | 26950000 | 30400000 | 3450000 | 0.017 |
| DMR5:53365301;DMR5:53490201;DMR5:53560501;DMR5:54589001;DMR5:54610101;DMR5:57026201;DMR5:58011201;DMR5:58386501 | 5 | 51600000 | 58950000 | 7350000 | 1.67E-07 |
| DMR8:11860301;DMR8:12439401;DMR8:13603601 | 8 | 11650000 | 13850000 | 2200000 | 0.017 |
| DMR8:22901801;DMR8:23883401;DMR8:24123401;DMR8:24651101;DMR8:24810801;DMR8:24850501;DMR8:26306401 | 8 | 22150000 | 26750000 | 4600000 | 2.63E-11 |
| DMR9:19526001;DMR9:20093101;DMR9:20439401 | 9 | 18450000 | 21500000 | 3050000 | 0.017 |
| DMR9:24201901;DMR9:25227801;DMR9:25731701;DMR9:26210101;DMR9:27015201 | 9 | 23750000 | 27650000 | 3900000 | 0.00014 |
| DMRZ:38648801;DMRZ:38745301;DMRZ:38864501;DMRZ:39510601;DMRZ:39662301;DMRZ:40335601;DMRZ:40862001 | Z | 36900000 | 41600000 | 4700000 | 2.63E-11 |
