# Supplementary material for: Epigenetic variation between urban and rural populations of Darwin’s finches
Source: BMC Evol Biol. 2017 Aug 24;17:183. doi: 10.1186/s12862-017-1025-9 (PMC5569522; doi:10.1186/s12862-017-1025-9)
Supplement: Supplementary file 7 — Gene associations with DMR detected in G. fortis sperm (A) and erythrocytes (B) and G. fuliginosa sperm (C) and erythrocytes (D). Description includes DMR name, gene symbol, entrez gene identification, chromosome number, start position site, ensemble gene identification number, gene description and gene classification category. (PDF 225 kb) [file 12862_2017_1025_MOESM7_ESM.pdf]

Supplemental Table S5A *G. fortis* **Multiple-Window Sperm DMR Gene Associations**

DMR Name	Gene Symbol	entrezgene	Chr	start_position	Ensembl #	Gene Description	Classification Category
DMR1:89900301	ENO2	100231650	1	89898643	ENSTGUG00000013292	enolase 2 (gamma - neuronal)	Metabolism
DMR1:89900301	LRRC23	NA	1	89846656	ENSTGUG00000018128	leucine rich repeat containing 23	Unknown
DMR1A:9608801	HGF	NA	1A	9584500	ENSTGUG00000002584	hepatocyte growth factor (hepapoietin A; scatter factor)	Growth Factor
DMR1A:33384201	MSRB3	100222850	1A	33347429	ENSTGUG00000006603	methionine sulfoxide reductase B3	Metabolism
DMR2:1016201		NA	2	992603	ENSTGUG00000000185	Uncharacterized protein	Unknown
DMR2:1016201	CCDC12	100228451	2	1006110	ENSTGUG00000000186	coiled-coil domain containing 12	Transcription
DMR2:11077401	NA	100232012	2	11020995	ENSTGUG00000000783	Uncharacterized protein	Unknown
DMR2:57355101	RPL15	100190572	2	57352106	ENSTGUG00000003325	ribosomal protein L15	Translation
DMR2:152511201	NA	NA	2	152312910	ENSTGUG00000012639	Uncharacterized protein	Unknown
DMR2:155551401	RHPN1	100230992	2	155541405	ENSTGUG00000012739	rhophilin - Rho GTPase binding protein 1	Signaling
DMR3:19616201	GALNT14	100229575	3	19573963	ENSTGUG00000004629	polypeptide N-acetylgalactosaminyltransferase 14	Golgi
DMR3:22144501	NRXN1	100228660	3	22119248	ENSTGUG00000005731	neurexin 1	Receptor
DMR3:82421001	HTR1B	751996	3	82421033	ENSTGUG00000012613	5-hydroxytryptamine (serotonin) receptor 1B - G protein-coupled	Receptor
DMR3:110673201	PAQR8	100220736	3	110674600	ENSTGUG00000013277	progestin and adipoQ receptor family member VIII	Receptor
DMR3:111805401	MSRA	NA	3	111667595	ENSTGUG00000013321	methionine sulfoxide reductase A	Metabolism
DMR3:112515501	FZD3	NA	3	112498936	ENSTGUG00000013332	frizzled class receptor 3	Receptor
DMR4:6462901	FAT4	100222872	4	6366597	ENSTGUG00000002042	FAT atypical cadherin 4	Receptor
DMR4:10513201	LRBA	100226445	4	10348610	ENSTGUG00000002514	LPS-responsive vesicle trafficking - beach and anchor containing	Transport
DMR4:58976401	JAKMIP1	101233368	4	58954507	ENSTGUG00000010025	janus kinase and microtubule interacting protein 1	Signaling
DMR4:67704101	CTNNA2	100221417	4	67538300	ENSTGUG00000010908	catenin (cadherin-associated protein) - alpha 2	Signaling
DMR4:67704101	LRRTM1	100218569	4	67703594	ENSTGUG00000010929	leucine rich repeat transmembrane neuronal 1	Receptor
DMR4A:4743001	RPS6KA6	100217905	4A	4707076	ENSTGUG00000002510	ribosomal protein S6 kinase - 90kDa - polypeptide 6	Signaling
DMR4A:19342701	CNGA2	NA	4A	19342297	ENSTGUG00000006426	cyclic nucleotide gated channel alpha 2]	Transport
DMR5:6667801	TMEM132A	NA	5	6667834	ENSTGUG00000006351	transmembrane protein 132A	Unknown
DMR5:6667801	CD6	NA	5	6674726	ENSTGUG00000006362	CD6 molecule	Receptor
DMR5:10950801	SOX6	100225773	5	10806780	ENSTGUG00000008522	SRY (sex determining region Y)-box 6	Transcription
DMR5:14025601	SYT8	100232540	5	14010649	ENSTGUG00000009405	synaptotagmin VIII	Transport
DMR6:3861701	SGMS1	100229900	6	3861953	ENSTGUG00000005065	sphingomyelin synthase 1	Metabolism
DMR6:21155001	NA	NA	6	21137374	ENSTGUG00000009180	Uncharacterized protein	Unknown
DMR7:1620001	HDAC4	100222021	7	1612998	ENSTGUG00000003197	histone deacetylase 4	Epigenetic
DMR8:24996701	NFIA	100221182	8	24945852	ENSTGUG00000009745	nuclear factor I/A	Transcription
DMR9:23196601	NA	NA	9	23120500	ENSTGUG00000011013	Uncharacterized protein	Unknown
DMR10:2121101	PLEC	NA	10	2115301	ENSTGUG00000004052	plectin	Cytoskeleton
DMR10:14143801	NA	100217734	10	14143898	ENSTGUG00000008405	Uncharacterized protein	Unknown
DMR10:14466301	SV2B	100222606	10	14442653	ENSTGUG00000008436	synaptic vesicle glycoprotein 2B	Protein Interaction
DMR10:17182001		NA	10	17184323	ENSTGUG00000008635	Uncharacterized protein	Unknown
DMR10:17182001	IGF1R	100227374	10	17184337	ENSTGUG00000008636	insulin-like growth factor 1 receptor	Receptor
DMR11:16380101	NA	NA	11	16377691	ENSTGUG00000009150	Uncharacterized protein	Unknown
DMR12:4785401	MST1R	NA	12	4785348	ENSTGUG00000005035	macrophage stimulating 1 receptor	Receptor
DMR12:5901001	NA	NA	12	5885739	ENSTGUG00000005918	Uncharacterized protein	Unknown
DMR13:11920001	GABRB2	100227722	13	11919977	ENSTGUG00000001428	gamma-aminobutyric acid (GABA) A receptor - beta 2	Receptor
DMR14:1181901	RAB40C	NA	14	1176454	ENSTGUG00000003408	RAB40C - member RAS oncogene family	Signaling
DMR14:3090701	RHBDF1	100230264	14	3088544	ENSTGUG00000004429	rhomboid 5 homolog 1 (Drosophila)	Protease
DMR15:2627801	NA	NA	15	2627887	ENSTGUG00000005468	Uncharacterized protein	Unknown
DMR15:2915401	IFT81	100224782	15	2910879	ENSTGUG00000005815	intraflagellar transport 81	Unknown
DMR17:3431501	NA	NA	17	3419406	ENSTGUG00000003291	Uncharacterized protein	Unknown
DMR18:991001		100190374	18	897346	ENSTGUG00000002914	Uncharacterized protein	Unknown
DMR18:991001	BTBD17	100225927	18	991544	ENSTGUG00000002937	BTB (POZ) domain containing 17	Unknown
DMR18:1081001	SLC38A10	NA	18	1064419	ENSTGUG00000002960	solute carrier family 38 - member 10	Transport
DMR18:1084101	SLC38A10	NA	18	1064419	ENSTGUG00000002960	solute carrier family 38 - member 10	Transport
DMR19:514001	RNF43	NA	19	494931	ENSTGUG00000003301	ring finger protein 43	Unknown
DMR19:5685501	RFFL	101233851	19	5681157	ENSTGUG00000004921	ring finger and FYVE-like domain containing E3 ubiquitin protein ligase	Proteolysis

DMR20:553601	RALGAPB	100224256	20	523559	ENSTGUG00000003151	Ral GTPase activating protein - beta subunit (non-catalytic)	Signaling
DMR20:1139801	NA	100230956	20	1136740	ENSTGUG00000003444	Uncharacterized protein	Unknown
DMR20:13641501	NA	NA	20	13633879	ENSTGUG00000008516	Uncharacterized protein	Unknown
DMR21:3356301	PLCH2	NA	21	3357561	ENSTGUG00000002915	phospholipase C - eta 2	Signaling
DMR23:4735301	EPHA10	NA	23	4734790	ENSTGUG00000001509	EPH receptor A10	Receptor
DMR25:797501	NA	100219169	25	797650	ENSTGUG00000004304	Uncharacterized protein	Unknown
DMR25:797501	NA	100222004	25	797650	ENSTGUG00000004304	Uncharacterized protein	Unknown
DMR27:383501	DDX42	100217897	27	383542	ENSTGUG00000001931	DEAD (Asp-Glu-Ala-Asp) box helicase 42	Translation
DMR27:4437801	NA	NA	27	4277732	ENSTGUG00000003306	Uncharacterized protein	Unknown
DMR27:4437801	NA	NA	27	4317779	ENSTGUG00000003320	Uncharacterized protein	Unknown
DMR28:4853401	KLHL26	100230034	28	4852037	ENSTGUG00000001064	kelch-like family member 26	Unknown
DMRUn:38440401	ARHGAP39	100223465	Un	38437090	ENSTGUG00000015026	Rho GTPase activating protein 39	Signaling
DMRUn:70786601	SYCP1	NA	Un	70782305	ENSTGUG00000015209	synaptonemal complex protein 1	Cell Cycle
DMRUn:70786601		NA	Un	70765920	ENSTGUG00000015195	Uncharacterized protein	Unknown
DMRUn:70799501	SYCP1	NA	Un	70782305	ENSTGUG00000015209	synaptonemal complex protein 1	Cell Cycle
DMRUn:70799501		NA	Un	70765920	ENSTGUG00000015195	Uncharacterized protein	Unknown
DMRUn:116431501	DNMT1	NA	Un	116426172	ENSTGUG00000013708	DNA (cytosine-5-)-methyltransferase 1	Epigenetic
DMRUn:116729701	DOCK7	NA	Un	116727282	ENSTGUG00000013835	Uncharacterized protein	Signaling
DMRUn:132241501	USP21	100231115	Un	132242124	ENSTGUG00000016056	ubiquitin specific peptidase 21	Proteolysis
DMRZ:38519001		NA	Z	38512069	ENSTGUG00000001724	Uncharacterized protein	Unknown
DMRZ:38519001	RPP25	NA	Z	38519149	ENSTGUG00000001727	ribonuclease P/MRP 25kDa subunit	Unknown

Supplemental Table S5B **_G. fortis_ Multiple-Window Erythrocyte DMR Gene Associations**

DMR Name	Gene Symbol	entrezgene	Chr	start_position	Ensemb #	Gene Description	Classification Category
DMR1:2682401	NA	NA	1	2421764	ENSTGUG00000005151	Uncharacterized protein	Unknown
DMR1:5421101	CXorf36	100227072	1	5410116	ENSTGUG00000006067	chromosome X open reading frame 36	Unknown
DMR1:17714201	GEMIN8	NA	1	17700218	ENSTGUG00000008162	gem (nuclear organelle) associated protein 8	Translation
DMR1:52621901	NA	100225399	1	52426258	ENSTGUG00000011857	Uncharacterized protein	Unknown
DMR1:59413201	NA	100219571	1	59285996	ENSTGUG00000012398	Uncharacterized protein	Unknown
DMR1:68474101	PIBF1	100218618	1	68431741	ENSTGUG00000012511	progesterone immunomodulatory binding factor 1	Immune
DMR1:83012401	NA	100228758	1	82998831	ENSTGUG00000012920	Malic enzyme	Metabolism
DMR1:93094801	NA	100219809	1	93077213	ENSTGUG00000013396	Uncharacterized protein	Unknown
DMR1A:14851501	CPNE8	NA	1A	14799211	ENSTGUG00000003596	copine VIII	Protein Interaction
DMR1A:46028501	IKBIP	NA	1A	46028147	ENSTGUG00000008755	IKBKB interacting protein	Protein Interaction
DMR1A:47095601	NA	100232057	1A	47068604	ENSTGUG00000009105	Uncharacterized protein	Unknown
DMR1A:70257101	MICAL3	NA	1A	70210553	ENSTGUG00000012499	microtubule associated monooxygenase - calponin and LIM domain containing 3	Unknown
DMR2:44270401	FARS2	NA	2	44124991	ENSTGUG00000002269	phenylalanyl-tRNA synthetase 2 - mitochondrial	Translation
DMR2:148037901	NA	NA	2	148037992	ENSTGUG00000012535	Uncharacterized protein	Unknown
DMR3:35391201	CRIM1	NA	3	35362730	ENSTGUG00000008626	cysteine rich transmembrane BMP regulator 1 (chordin-like)	Development
DMR5:2884401	ELP4	NA	5	2764406	ENSTGUG00000004816	elongator acetyltransferase complex subunit 4	Transcription
DMR5:17085201	NA	NA	5	17073049	ENSTGUG00000010089	Amino acid transporter	Transport
DMR6:4069801	NA	NA	6	3961424	ENSTGUG00000005123	Uncharacterized protein	Unknown
DMR6:4069801	NA	NA	6	3970891	ENSTGUG00000005129	Uncharacterized protein	Unknown
DMR6:15841201	GBF1	100227027	6	15830427	ENSTGUG00000007414	golgi brefeldin A resistant guanine nucleotide exchange factor 1	Signaling
DMR6:22127301	FBXW4	100229466	6	21890389	ENSTGUG00000009903	F-box and WD repeat domain containing 4	Unknown
DMR6:22127301	FGF8	NA	6	22049471	ENSTGUG00000009956	fibroblast growth factor 8 (androgen-induced)	Growth Factor
DMR6:24244501	SORCS3	100229522	6	24203189	ENSTGUG00000010448	sortilin-related VPS10 domain containing receptor 3	Receptor
DMR7:7103301	MRAS	100225544	7	7083869	ENSTGUG00000004727	muscle RAS oncogene homolog	Signaling
DMR8:9281501	PTBP2	100219685	8	9249676	ENSTGUG00000005558	polypyrimidine tract binding protein 2	Translation
DMR12:10036401	ABTB1	100223102	12	10018786	ENSTGUG00000008002	ankyrin repeat and BTB (POZ) domain containing 1	Unknown
DMR12:20282601	ITPR1	NA	12	20139304	ENSTGUG00000010241	inositol 1 -4 -5-trisphosphate receptor - type 1	Receptor
DMR13:246201	STK32A	NA	13	246166	ENSTGUG00000000062	serine/threonine kinase 32A	Signaling
DMR13:11691501	NA	100227780	13	11677740	ENSTGUG00000001419	Phospholipid-transporting ATPase	Transport
DMR14:10140501	CACNA1H	NA	14	10031279	ENSTGUG00000006881	calcium channel - voltage-dependent - T type - alpha 1H subunit	Transport
DMR14:10140501	B9D1	NA	14	10093952	ENSTGUG00000007019	B9 protein domain 1 [	Extracellular Matrix
DMR15:5515601	MN1	100223802	15	5486760	ENSTGUG00000007793	meningioma (disrupted in balanced translocation) 1	Unknown
DMR15:12868101	NA	NA	15	12853825	ENSTGUG00000010495	Uncharacterized protein	Unknown
DMR15:14137901	AIFM3	100227335	15	14135686	ENSTGUG00000010632	apoptosis-inducing factor - mitochondrion-associated - 3	Apoptosis
DMR18:7751401	ST6GALNAC2	100224588	18	7751042	ENSTGUG00000007819	ST6 (alpha-N-acetyl-neuraminyl-2 -3-beta-galactosyl-1 -3)-N-acetylgalactosaminide alpha-2 -6-sialyltransferase 2	Gogli
DMR19:9135801	NA	NA	19	9132699	ENSTGUG00000007767	Uncharacterized protein	Unknown
DMR20:15138501	KCNB1	NA	20	15135879	ENSTGUG00000008781	potassium channel - voltage gated Shab related subfamily B - member 1	Transport
DMR21:4415901	ACAP3	NA	21	4404694	ENSTGUG00000003217	ArfGAP with coiled-coil - ankyrin repeat and PH domains 3	Signaling
DMR26:3225201	RAP1A	100151697	26	3158273	ENSTGUG00000001456	Taeniopygia guttata RAP1A - member of RAS oncogene family (RAP1A) - mRNA.	Signaling
DMRUn:115914401	NA	NA	Un	115904314	ENSTGUG00000016835	Uncharacterized protein	Unknown
DMRUn:167343801	NA	NA	Un	167343490	ENSTGUG00000016875	Uncharacterized protein	Unknown
DMRZ:45691301	ISL1	100231918	Z	45690056	ENSTGUG00000002375	ISL LIM homeobox 1	Transcription
DMRZ:70647701	EDIL3	100232785	Z	70483471	ENSTGUG00000006826	EGF-like repeats and discoidin I-like domains 3	Development

Supplemental Table S5C ***G. fuliginosa*** **Multiple-Window Sperm DMR Gene Associations**

DMR Name	Gene Symbol	entrezgene	Chr	start_position	Ensembl #	Gene Description	Classification Category
DMR1:10654201	IL1RAPL1	100219378	1	10652160	ENSTGUG00000007085	interleukin 1 receptor accessory protein-like 1	Receptor
DMR1:80766701	FAT3	100223418	1	80634098	ENSTGUG00000012873	FAT atypical cadherin 3	Extracellular Matrix (ECM)
DMR1A:11577701	PTPN12	NA	1A	11558479	ENSTGUG00000002717	protein tyrosine phosphatase - non-receptor type 12	Signaling
DMR1A:24114101	WNT2	100221216	1A	24114687	ENSTGUG00000005016	wingless-type MMTV integration site family member 2	Growth Factor
DMR1A:27064701	IMMP2L	100226070	1A	26702220	ENSTGUG00000005588	IMP2 inner mitochondrial membrane peptidase-like (S. cerevisiae)	Proteolysis
DMR1A:29521501	TMEM117	100228057	1A	29424286	ENSTGUG00000005962	transmembrane protein 117	Unknown
DMR1A:37122201	NA	NA	1A	37109977	ENSTGUG00000007370	Uncharacterized protein	Unknown
DMR1A:48389101	TTC26	100225372	1A	48361671	ENSTGUG00000009550	tetratricopeptide repeat domain 26	Unknown
DMR1A:59545101	KDM7A	100223451	1A	59545795	ENSTGUG00000011686	lysine (K)-specific demethylase 7A	Epigenetic
DMR1A:61248301	C2CD5	NA	1A	61201716	ENSTGUG00000011766	C2 calcium-dependent domain containing 5	Unknown
DMR2:7094701	GALNT11	100231024	2	7087083	ENSTGUG00000000608	polypeptide N-acetylgalactosaminyltransferase 11	Gogli
DMR2:19980701	NA	NA	2	19983441	ENSTGUG00000001217	Uncharacterized protein	Unknown
DMR2:21459501	NA	NA	2	21459108	ENSTGUG00000001259	Uncharacterized protein	Unknown
DMR2:57223201	NA	NA	2	57030969	ENSTGUG00000003303	Uncharacterized protein	Unknown
DMR2:60537101	OSBPL10	100222583	2	60501996	ENSTGUG00000003656	oxysterol binding protein-like 10	Binding Protein
DMR2:94031401	NA	NA	2	93945597	ENSTGUG00000008651	Uncharacterized protein	Unknown
DMR2:118325701	TOX	100232611	2	118235600	ENSTGUG00000011174	thymocyte selection-associated high mobility group box	Immune
DMR2:134150201	NIPAL2	NA	2	134144014	ENSTGUG00000012018	NIPA-like domain containing 2	Development
DMR2:144740601	FBXO32	100224944	2	144729030	ENSTGUG00000012442	F-box protein 32	Transcription
DMR3:14233101	UBR2	100225781	3	14191156	ENSTGUG00000003261	ubiquitin protein ligase E3 component n-recognin 2	Proteolysis
DMR3:20417201	LCLAT1	NA	3	20382321	ENSTGUG00000005391	lysocardiolipin acyltransferase 1	Metabolism
DMR3:37439001	PARK2	100221229	3	37384893	ENSTGUG00000009367	parkin RBR E3 ubiquitin protein ligase	Proteolysis
DMR3:41905401	DLL1	100230776	3	41897719	ENSTGUG00000009780	delta-like 1 (Drosophila)	Receptor
DMR3:43634901	SIPA1L2	100228885	3	43583239	ENSTGUG00000010103	signal-induced proliferation-associated 1 like 2	Signaling
DMR3:53617101	SNX9	100225096	3	53592751	ENSTGUG00000011096	sorting nexin 9	Transport
DMR3:78687001	AKIRIN2	NA	3	78683482	ENSTGUG00000012437	akirin 2	Unknown
DMR3:85093801	FAM135A	100227848	3	85067721	ENSTGUG00000012721	family with sequence similarity 135 - member A	Unknown
DMR3:89692801	BMP5	100232671	3	89677349	ENSTGUG00000012830	bone morphogenetic protein 5	Growth Factor
DMR3:92570201	CSMD1	100230644	3	92239007	ENSTGUG00000012896	CUB and Sushi multiple domains 1	Signaling
DMR3:95496201	PXDN	100230769	3	95422869	ENSTGUG00000012962	peroxidasin	Metabolism
DMR4:30010001	RAPGEF2	100224541	4	30007643	ENSTGUG00000005715	Rap guanine nucleotide exchange factor (GEF) 2	Signaling
DMR4A:4645401	HDX	100223075	4A	4645348	ENSTGUG00000002499	highly divergent homeobox	Unknown
DMR5:17116201	NA	NA	5	17093478	ENSTGUG00000010095	Amino acid transporter	Transport
DMR5:20105501	EXT2	100231605	5	20077856	ENSTGUG00000010249	exostosin glycosyltransferase 2	Gogli
DMR5:22209801	AMBRA1	NA	5	22154488	ENSTGUG00000010628	autophagy/beclin-1 regulator 1	Apoptosis
DMR5:25054401	UBR1	NA	5	25031883	ENSTGUG00000011165	ubiquitin protein ligase E3 component n-recognin 1	Proteolysis
DMR5:29800501	RYR3	100219104	5	29645499	ENSTGUG00000011653	ryanodine receptor 3	Receptor
DMR6:5313901	C10orf107	NA	6	5284088	ENSTGUG00000005288	chromosome 10 open reading frame 107	Unknown
DMR6:28395601	NA	NA	6	28226525	ENSTGUG00000010983	Uncharacterized protein	Unknown
DMR7:2821801	IQCA1	NA	7	2756467	ENSTGUG00000003628	IQ motif containing with AAA domain 1	Unknown
DMR7:27843701	ZNF804A	100221331	7	27813179	ENSTGUG00000011155	zinc finger protein 804A	Unknown
DMR7:32399701	NA	NA	7	32359427	ENSTGUG00000011682	Uncharacterized protein	Unknown
DMR8:25817401	NA	100218377	8	25813953	ENSTGUG00000009906	Uncharacterized protein	Unknown
DMR9:15033901	MB21D2	100221081	9	15029216	ENSTGUG00000009252	Mab-21 domain containing 2	Unknown
DMR10:8862401	TMOD2	100225462	10	8857237	ENSTGUG00000006888	tropomodulin 2 (neuronal)	Cytoskeleton
DMR11:17270401	CDH8	NA	11	17141697	ENSTGUG00000009281	cadherin 8 - type 2	Extracellular Matrix (ECM)
DMR14:15125101	NA	100220325	14	15124742	ENSTGUG00000008831	Ubiquitin carboxyl-terminal hydrolase	Proteolysis
DMR15:8420501	TOP3B	NA	15	8411299	ENSTGUG00000008644	topoisomerase (DNA) III beta	Transcription

DMR19:8815901	GDPD1	100224315	19	8812582	ENSTGUG00000007493	glycerophosphodiester phosphodiesterase domain containing 1	Metabolism
DMR26:1999201	KLHL12	NA	26	1977574	ENSTGUG00000001234	kelch-like family member 12	Cytoskeleton
DMRUn:152715201	NA	NA	Un	152582138	ENSTGUG00000018382	Uncharacterized protein	Unknown
DMRZ:58234701	DENND4C	100230179	Z	58237261	ENSTGUG00000004350	DENN/MADD domain containing 4C	Unknown

Supplemental Table S5D *G. fuliginosa* **Multiple-Window Erythrocyte DMR Gene Associations**

DMR Name	Gene Symbol	entrezgene	Chr	start_position	ensembl_gene_id	Gene Description	Classification Category
DMR1:15514901	NA	NA	1	15501404	ENSTGUG00000007781	Uncharacterized protein	Unknown
DMR1:30194301	RNF149	100221793	1	30188297	ENSTGUG00000009945	ring finger protein 149	Proteolysis
DMR1:49804201	NA	NA	1	49704389	ENSTGUG00000011664	Uncharacterized protein	Unknown
DMR1:69585701	LMO7	NA	1	69547644	ENSTGUG00000012539	LIM domain 7	Cytoskeleton
DMR1:79976701	NA	100229192	1	79958222	ENSTGUG00000012829	Uncharacterized protein	Unknown
DMR1:80849001	FAT3	100223418	1	80634098	ENSTGUG00000012873	FAT atypical cadherin 3	ECM
DMR1:94822701	LSAMP	NA	1	94669017	ENSTGUG00000013402	limbic system-associated membrane protein	ECM
DMR1A:536501	NET1	100228711	1A	528641	ENSTGUG00000001945	neuroepithelial cell transforming 1	Signaling
DMR1A:3291201	SFMBT2	100218182	1A	3209575	ENSTGUG00000002052	Scm-like with four mbt domains 2	Epigenetic
DMR1A:25828501	PPP1R3A	NA	1A	25815970	ENSTGUG00000005399	protein phosphatase 1 - regulatory subunit 3A	Signaling
DMR1A:38048601	NAV3	100229468	1A	38040492	ENSTGUG00000007519	neuron navigator 3	Development
DMR1A:50021401	CACNA1I	NA	1A	50003360	ENSTGUG00000010198	calcium channel - voltage-dependent - T type - alpha 1I subunit	Transport
DMR1A:60465701	SOX5	100220574	1A	60317901	ENSTGUG00000011749	SRY (sex determining region Y)-box 5	Transcription
DMR1A:67777301	PLEKHA5	NA	1A	67772119	ENSTGUG00000012354	pleckstrin homology domain containing - family A member 5	Signaling
DMR2:21606201	NA	NA	2	21604293	ENSTGUG00000001271	Uncharacterized protein	Unknown
DMR2:66551001	EEPD1	NA	2	66510181	ENSTGUG00000005472	endonuclease/exonuclease/phosphatase family domain containing 1	Signaling
DMR2:84797701	MYH6	NA	2	84795322	ENSTGUG00000007833	myosin - heavy chain 6 - cardiac muscle - alpha	Cytoskeleton
DMR2:88439501	FHOD3	NA	2	88300265	ENSTGUG00000007999	formin homology 2 domain containing 3	Unknown
DMR2:88582201	C18orf21	100218608	2	88579126	ENSTGUG00000008036	chromosome 18 open reading frame 21	Unknown
DMR2:102792501	GNAL	100219216	2	102746499	ENSTGUG00000009707	guanine nucleotide binding protein (G protein) - alpha activating activity polypeptide - olfactory type	Signaling
DMR2:109112201	OSBPL1A	100223128	2	109039838	ENSTGUG00000010525	oxysterol binding protein-like 1A	Binding Protein
DMR2:110670701	CDH2	100229697	2	110571137	ENSTGUG00000010620	cadherin 2 - type 1 - N-cadherin (neuronal)	ECM
DMR2:121488201	NA	100231693	2	121486449	ENSTGUG00000011308	Uncharacterized protein	Unknown
DMR2:132400801	CDH17	NA	2	132378941	ENSTGUG00000011871	cadherin 17 - LI cadherin (liver-intestine)	ECM
DMR2:138528101	NA	100223993	2	138507126	ENSTGUG00000012216	Uncharacterized protein	Unknown
DMR2:141908201	EIF3H	100190645	2	141896849	ENSTGUG00000012302	eukaryotic translation initiation factor 3 - subunit H	Translation
DMR2:151126601	FAM135B	100227463	2	151114975	ENSTGUG00000012621	family with sequence similarity 135 - member B	Unknown
DMR2:151971901	KCNK9	100230281	2	151900356	ENSTGUG00000012636	potassium channel - two pore domain subfamily K - member 9	Transport
DMR2:155785401	ZC3H3	NA	2	155762072	ENSTGUG00000012743	zinc finger CCCH-type containing 3	Translation
DMR3:5223801	TASP1	NA	3	5146840	ENSTGUG00000002456	taspase - threonine aspartase - 1	Proteolysis
DMR3:21546801	FOXN3	NA	3	21548956	ENSTGUG00000005623	forkhead box N3	Unknown
DMR3:47496101	UST	100218364	3	47457997	ENSTGUG00000010673	uronyl-2-sulfotransferase	Metabolism
DMR3:54732001	NOX3	100228883	3	54716130	ENSTGUG00000011124	NADPH oxidase 3	Metabolism
DMR3:70631201	PRDM1	100219562	3	70624594	ENSTGUG00000012204	PR domain containing 1 - with ZNF domain	Transcription
DMR3:89074901	PRIM2	100228796	3	88989762	ENSTGUG00000012790	primase - DNA - polypeptide 2 (58kDa)	Metabolism
DMR3:89714201	BMP5	100232671	3	89677349	ENSTGUG00000012830	bone morphogenetic protein 5	Growth Factor
DMR3:90528101	GCLC	100218278	3	90521029	ENSTGUG00000012854	glutamate-cysteine ligase - catalytic subunit	Metabolism
DMR3:110489001	NA	NA	3	110359582	ENSTGUG00000013268	Uncharacterized protein	Unknown
DMR4:5567801	SPATA5	100221993	4	5549460	ENSTGUG00000002029	spermatogenesis associated 5	Development
DMR4:6440901	FAT4	100222872	4	6366597	ENSTGUG00000002042	FAT atypical cadherin 4	Signaling
DMR4:11522601	ARHGAP10	100227425	4	11426980	ENSTGUG00000002594	Rho GTPase activating protein 10	Signaling
DMR4:23287201	LEF1	100228376	4	23247602	ENSTGUG00000004013	lymphoid enhancer-binding factor 1	Transcription
DMR4:30011901	RAPGEF2	100224541	4	30007643	ENSTGUG00000005715	Rap guanine nucleotide exchange factor (GEF) 2	Signaling
DMR4:38514801	ENPP6	100225148	4	38502012	ENSTGUG00000006653	ectonucleotide pyrophosphatase/phosphodiesterase 6	Signaling

DMR4:47123001	NA	100225702	4	47122672	ENSTGUG00000008603	Uncharacterized protein	Unknown
DMR4A:9445601	MCTS1	100190027	4A	9359760	ENSTGUG00000003338	malignant T cell amplified sequence 1	Receptor
DMR4A:9445601	AKAP14	100218817	4A	9443728	ENSTGUG00000003440	A kinase (PRKA) anchor protein 14	Signaling
DMR5:143201	NELL1	100228095	5	75638	ENSTGUG00000004458	NEL-like 1 (chicken) [Source:HGNC Symbol;Acc:HGNC:7750]	Development
DMR5:7862001	NA	100224878	5	7862469	ENSTGUG00000007330	Uncharacterized protein	Unknown
DMR5:15807101	RPLP2	100221892	5	15762188	ENSTGUG00000009801	ribosomal protein - large - P2	Translation
DMR5:23337901	INO80	100227760	5	23289026	ENSTGUG00000010786	INO80 complex subunit	Transcription
DMR5:28566801	GPHN	NA	5	28455478	ENSTGUG00000011577	gephyrin	Receptor
DMR5:40106301	NA	NA	5	39895138	ENSTGUG00000012355	Uncharacterized protein	Unknown
DMR5:44895801	TTC7B	NA	5	44815864	ENSTGUG00000012501	tetratricopeptide repeat domain 7B	Metabolism
DMR5:48919901	NA	NA	5	48861713	ENSTGUG00000012709	Uncharacterized protein	Unknown
DMR5:53490201	CEP170B	NA	5	53432980	ENSTGUG00000012907	centrosomal protein 170B	Unknown
DMR5:54589001	BRF1	NA	5	54573873	ENSTGUG00000012926	BRF1 - RNA polymerase III transcription initiation factor 90 kDa subunit	Transcription
DMR5:54610101	BRF1	NA	5	54573873	ENSTGUG00000012926	BRF1 - RNA polymerase III transcription initiation factor 90 kDa subunit	Transcription
DMR5:58386501	TMEM260	100230578	5	58386478	ENSTGUG00000013057	transmembrane protein 260	Unknown
DMR6:25933601	NA	NA	6	25926844	ENSTGUG00000010665	Uncharacterized protein	Unknown
DMR7:10812401	SLC4A3	100223699	7	10813277	ENSTGUG00000006252	solute carrier family 4 (anion exchanger) - member 3	Transport
DMR7:11920001	IFIH1	NA	7	11915850	ENSTGUG00000006914	interferon induced with helicase C domain 1	Transcription
DMR7:26104901	ORMDL1	NA	7	26101329	ENSTGUG00000010880	ORMDL sphingolipid biosynthesis regulator 1	Metabolism
DMR8:4542101	CRB1	100223846	8	4494806	ENSTGUG00000004300	crumbs family member 1 - photoreceptor morphogenesis associated	Development
DMR8:10439801	RPL5	100190411	8	10438158	ENSTGUG00000005988	ribosomal protein L5	Translation
DMR8:10439801	SNORD21	NA	8	10440268	ENSTGUG00000018060	Small nucleolar RNA SNORD21	Unknown
DMR8:26306401	AK4	NA	8	26304563	ENSTGUG00000010010	adenylate kinase 4	Signaling
DMR9:5238901	NA	100221456	9	5228092	ENSTGUG00000006742	Uncharacterized protein	Unknown
DMR9:7457601	SPSB4	100225409	9	7410458	ENSTGUG00000007647	splA/ryanodine receptor domain and SOCS box containing 4	Unknown
DMR9:20093101	MFN1	100220202	9	20085820	ENSTGUG00000010693	mitofusin 1	Signaling
DMR9:25731701	MFSD1	100222737	9	25730655	ENSTGUG00000011199	major facilitator superfamily domain containing 1	Transport
DMR9:26210101	NA	NA	9	26202250	ENSTGUG00000011240	Uncharacterized protein	Unknown
DMR9:27015201	PFN2	NA	9	27012835	ENSTGUG00000011405	profilin 2	Cytoskeleton
DMR10:7027301	CGNL1	NA	10	6994325	ENSTGUG00000006242	cingulin-like 1	Cytoskeleton
DMR10:13256501	DET1	100217762	10	13250087	ENSTGUG00000008339	de-etiolated homolog 1 (Arabidopsis)	Proteolysis
DMR10:17985101	ALDH1A3	100231202	10	17976236	ENSTGUG00000008854	aldehyde dehydrogenase 1 family - member A3	Metabolism
DMR10:18730201	DENND4A	100229269	10	18728541	ENSTGUG00000009220	DENN/MADD domain containing 4A	Unknown
DMR11:10915301	BANP	NA	11	10820357	ENSTGUG00000007690	BTG3 associated nuclear protein	Transcription
DMR11:19202501	NA	NA	11	19195065	ENSTGUG00000009520	Uncharacterized protein	Unknown
DMR12:424701	NA	NA	12	347145	ENSTGUG00000003635	Uncharacterized protein	Unknown
DMR12:16143901	FAM19A1	100228414	12	16075500	ENSTGUG00000009669	family with sequence similarity 19 (chemokine (C-C motif)-like) - member A1	Cytokine
DMR13:4235701	EBF1	100228980	13	4019793	ENSTGUG00000000593	early B-cell factor 1	Transcription
DMR14:1865701	RAB11FIP3	NA	14	1810964	ENSTGUG00000003751	RAB11 family interacting protein 3 (class II)	Signaling
DMR14:7746201	MYH11	100230210	14	7725174	ENSTGUG00000005378	myosin - heavy chain 11 - smooth muscle	Cytoskeleton
DMR15:11293101	EIF4ENIF1	NA	15	11294615	ENSTGUG00000009790	eukaryotic translation initiation factor 4E nuclear import factor 1	Translation
DMR17:6864401	PRRC2B	NA	17	6861636	ENSTGUG00000004903	proline-rich coiled-coil 2B	Unknown
DMR17:11018301	PBX3	NA	17	10940614	ENSTGUG00000007364	pre-B-cell leukemia homeobox 3	Transcription
DMR18:1033201		100190374	18	897346	ENSTGUG00000002914	Uncharacterized protein	Unknown
DMR18:1033201	NPLOC4	NA	18	1020952	ENSTGUG00000002958	nuclear protein localization 4 homolog (S. cerevisiae)	Proteolysis
DMR18:8856901	MRPS7	NA	18	8759169	ENSTGUG00000008655	mitochondrial ribosomal protein S7	Translation
DMR19:10177801	ACACA	NA	19	10154218	ENSTGUG00000008074	acetyl-CoA carboxylase alpha	Metabolism
DMR20:12291301	NA	100220955	20	12289073	ENSTGUG00000008146	Uncharacterized protein	Unknown

DMR20:12433001	VAPB	NA	20	12407416	ENSTGUG00000008176	VAMP (vesicle-associated membrane protein)-associated protein B and C	Transport
DMR20:13029701	BMP7	NA	20	12994772	ENSTGUG00000008348	bone morphogenetic protein 7	Growth Factor
DMR21:3618001	SKI	NA	21	3567060	ENSTGUG00000002944	SKI proto-oncogene	Development
DMR21:3922101	NA	NA	21	3829014	ENSTGUG00000002984	Uncharacterized protein	Unknown
DMR21:4941801	UBE4B	NA	21	4917870	ENSTGUG00000003533	ubiquitination factor E4B	Proteolysis
DMR21:5494101	RERE	NA	21	5384095	ENSTGUG00000003924	arginine-glutamic acid dipeptide (RE) repeats	Unknown
DMR22:3011601	NA	NA	22	3005531	ENSTGUG00000005141	Uncharacterized protein	Unknown
DMR23:2525401	FAM76A	100232402	23	2514050	ENSTGUG00000000951	family with sequence similarity 76 - member A	Unknown
DMR24:1935801	NA	100227293	24	1915428	ENSTGUG00000000375	Uncharacterized protein	Unknown
DMR26:3370501	RAP1A	100151697	26	3158273	ENSTGUG00000001456	Taeniopygia guttata RAP1A - member of RAS oncogene family (RAP1A) - mRNA	Signaling
DMR26:4774101	NA	NA	26	4749296	ENSTGUG00000001836	Uncharacterized protein	Unknown
DMR27:3371401	TLK2	NA	27	3357401	ENSTGUG00000003073	tousled-like kinase 2	Signaling
DMR28:538701	NA	NA	28	507677	ENSTGUG00000000077	Uncharacterized protein	Unknown
DMRZ:8349501	NA	NA	Z	8261519	ENSTGUG00000000475	Uncharacterized protein	Unknown
DMRZ:33485401	SETBP1	100228402	Z	33303203	ENSTGUG00000001615	SET binding protein 1	Transcription
DMRZ:39662301	NA	NA	Z	39660746	ENSTGUG00000001811	Uncharacterized protein	Unknown
DMRZ:46606301	FST	100226154	Z	46604913	ENSTGUG00000002425	follistatin	Growth Factor
DMRZ:49883001	IPO11	100222001	Z	49853180	ENSTGUG00000002759	importin 11	Transport
DMRZ:66310201	FCHO2	100218413	Z	66303495	ENSTGUG00000005780	FCH domain only 2	Cytoskeleton
