# Supplementary material for: Epigenetic variation between urban and rural populations of Darwin’s finches
Source: BMC Evol Biol. 2017 Aug 24;17:183. doi: 10.1186/s12862-017-1025-9 (PMC5569522; doi:10.1186/s12862-017-1025-9)

Supplemental Figure S5

(A) Fuliginosa RBC Urban (U) Pairwise DMR Comparison

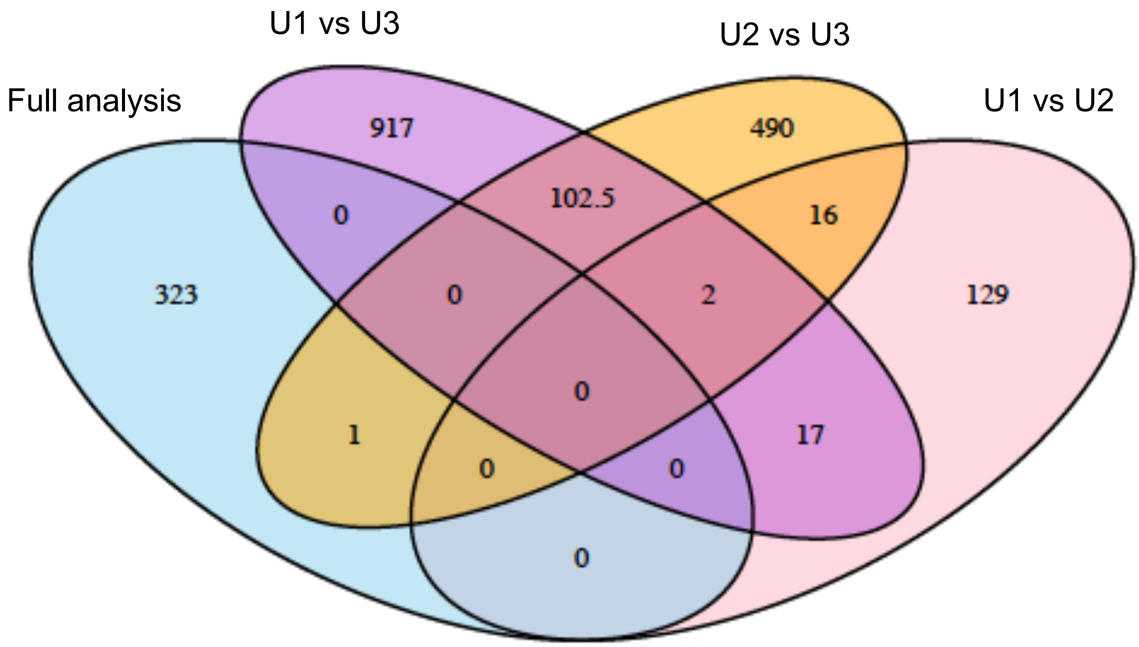

(B) Fuliginosa RBC Rural (R) Pairwise DMR Comparison

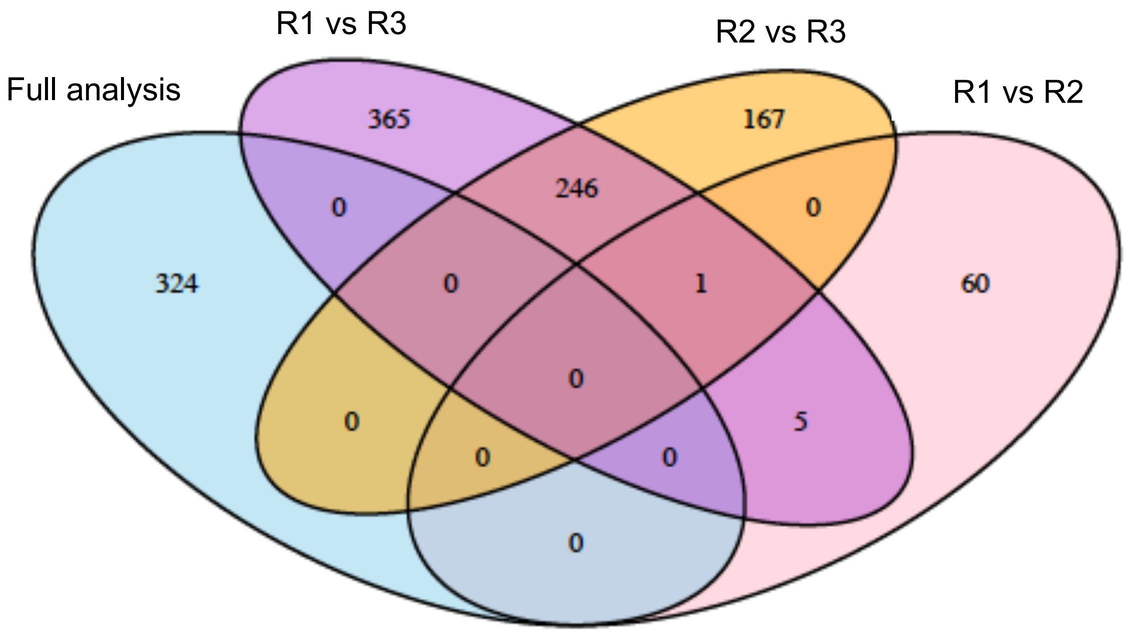

Supplemental Figure S5 (continued)

(C) Fortis RBC Urban (U) Pairwise DMR Comparison

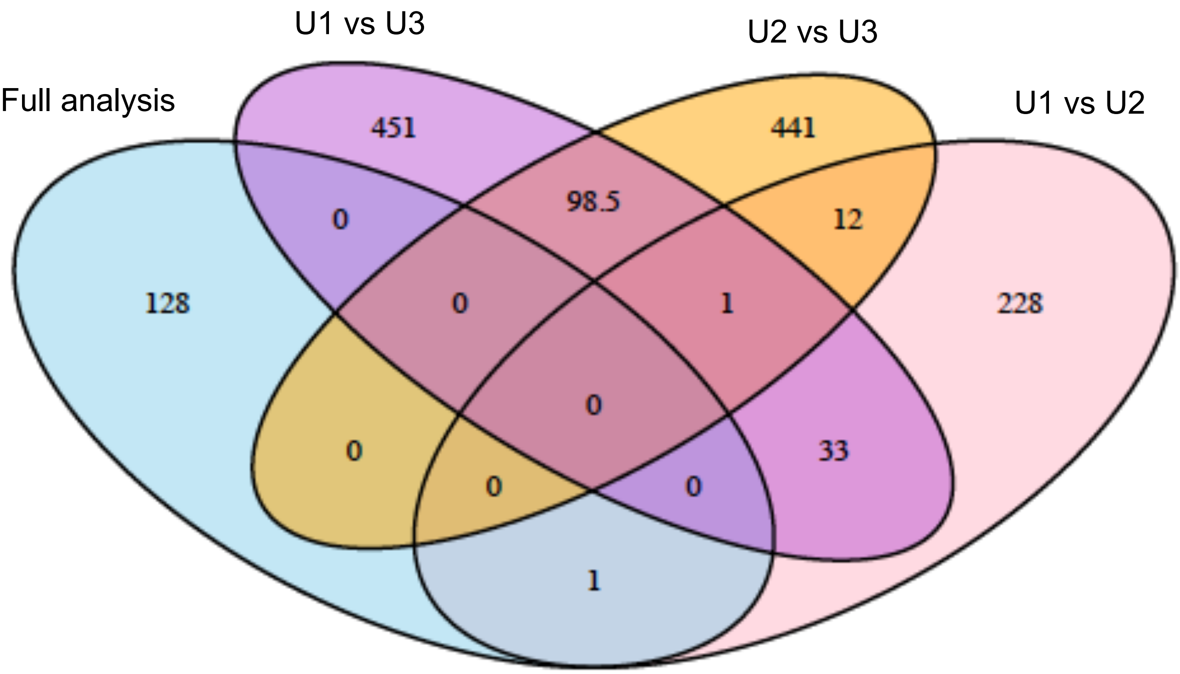

(D) Fortis RBC Rural (R) Pairwise DMR Comparison

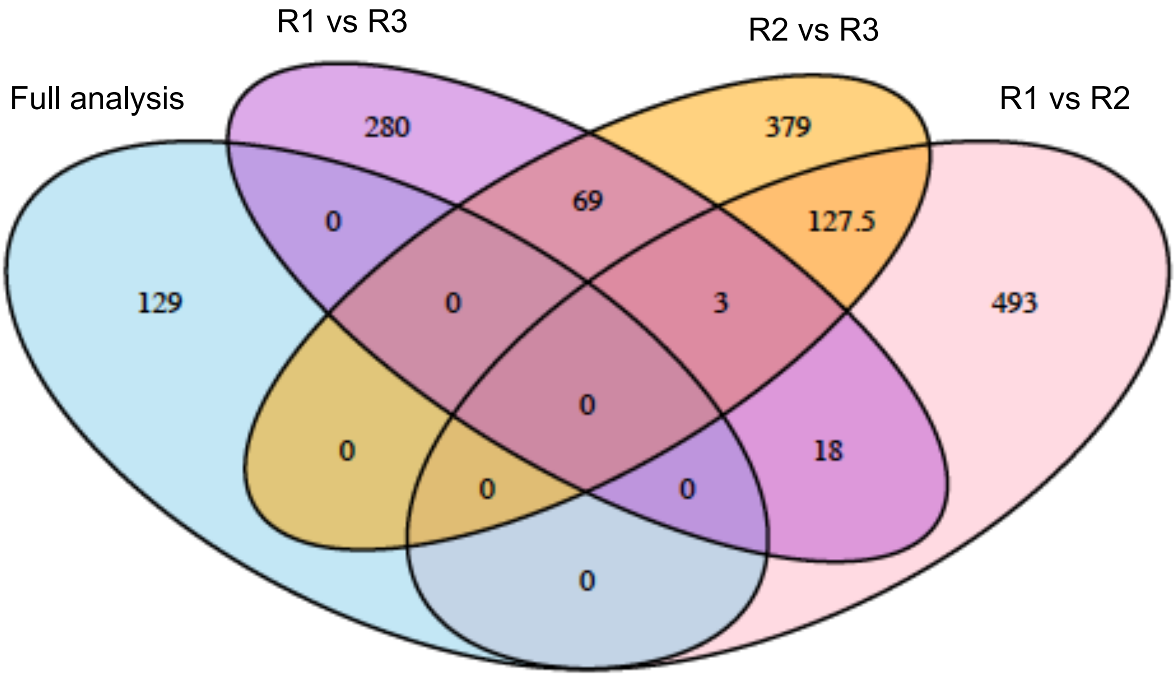

Supplement: Supplementary file 10 — DMRs identified in pairwise comparison of pools within populations: (A) G. Fuliginosa RBC urban analysis, (B) G. fuliginosa-RBC rural analysis, (C) G. fortis RBC urban analysis, and (D) G. fortis rural analysis. Numbers indicate DMRs between urban (U) or rural (R) individual pools (1-3). “Full analysis” are DMRs identified between urban and rural pools. DMRs identified in the full analysis were found independently of within-site variation. (PDF 98 kb) [file 12862_2017_1025_MOESM10_ESM.pdf]
